# Computational study of the structural ensemble of CC chemokine receptor type 5 (CCR5) and its interactions with different ligands

**DOI:** 10.1371/journal.pone.0275269

**Published:** 2022-10-17

**Authors:** Guillermo Goode-Romero, Laura Dominguez

**Affiliations:** Departamento de Fisicoquímica, Facultad de Química, Universidad Nacional Autónoma de México, Mexico City, Mexico; University of Michigan, UNITED STATES

## Abstract

CC Chemokine receptor 5 (CCR5), a member of the Superfamily of G Protein-Coupled Receptors (GPCRs), is an important effector in multiple physiopathological processes such as inflammatory and infectious entities, including central nervous system neuroinflammatory diseases such as Alzheimer’s disease, recovery from nervous injuries, and in the HIV-AIDS infective processes. Thus, CCR5 is an attractive target for pharmacological modulation. Since maraviroc was described as a CCR5 ligand that modifies the HIV-AIDS progression, multiple efforts have been developed to describe the functionality of the receptor. In this work, we characterized key structural features of the CCR5 receptor employing extensive atomistic molecular dynamics (MD) in its apo form and in complex with an endogenous agonist, the chemokine CCL5/RANTES, an HIV entry inhibitor, the partial inverse agonist maraviroc, and the experimental antagonists Compound 21 and 34, aiming to elucidate the structural features and mechanistic processes that constitute its functional states, contributing with structural details and a general understanding of this relevant system.

## Introduction

The chemokines are a subfamily of cytokines with chemotactic and communication activities, expressed and secreted by many cell types such as white blood cells, smooth-muscle, endothelial and neural-derived cells, in response to several stimuli. They are implicated in multiple physiological and pathological processes, including cell migration and recruitment, immune activation responses, acute inflammation, and survival or death induction of many cell types [1]. This family of small-proteins is classified according to their cysteine-motif in the primary sequences as CXC (or α-chemokines), CC (β-chemokines), XC (γ-chemokine or lymphotactin-1/2) and CX3C (δ-chemokine, CX3CL1 or fractalkine), where X denotes a variable residue. The principal chemokine receptors belong to the G Protein-Coupled Receptor (GPCR) superfamily. CCR5 serves as a receptor of multiple C-C chemokines, mainly CCL5, CCL8/MCP-2, and CCL3/MIP-1α, with important roles in inflammatory and neuroinflammatory processes seen in both acute and chronic, peripheral, and central damage. Like many other surface receptors, CCR5 is a receptor of external proteins, particularly of viral origin. Importantly, it acts as one of the most common coreceptors of the glycoprotein 120 of the spike from human immunodeficiency virus 1 (HIV-1) [2–4].

The CCL5 chemokine was first described in T-cell lymphocytes with cDNA libraries as the *R*egulated (increased production) upon *A*ctivation expressed by *N*ormal *T* c*e*lls (as well as other cells) and presumably (now confirmed) *S*ecreted factor (RANTES) [5]. Later, it was identified as a chemokine involved in multiple physiological and pathological signaling and processes. As the principal co-receptor for the HIV-1 virus, many efforts have been performed to target CCR5 to offer a small-molecule drug able to interfere with the HIV-1 infective process. The first marketed drug with blocking activity on CCR5, maraviroc (MRV), was obtained from a rational investigation and computer-aided development as a novel mechanism to fight the HIV-AIDS pandemic [6–8]. The CCR5-MRV complex was elucidated by X-ray crystallography, and a large series of CCR5 ligands have been under investigation [9]. Importantly, during the past ten years, the modulation of CCR5 has also been studied as a potential therapeutic target for central nervous system (CNS) pathologies, such as stroke and acute brain traumatic injury [10], autoimmune encephalomyelitis [11], acute injury of the spinal cord [12], some models of epilepsy in animals [13], metastatic cancer [14–16], and in COVID-19 disease [17]. In fact, CCR5, along with other chemokine receptors [18–27] have also been recognized as key participants in neurodegenerative diseases such as Alzheimer’s disease and other neuroinflammatory entities.

In this work, we employed extensive atomistic Molecular Dynamics (MD) simulations to describe the structural ensemble of CCR5 in its apo and ligand-bound forms and to characterize important CCR5 ligand-bound interactions that lead to CCR5 activation. This structural information will certainly be useful for developing treatments for such complex neurological diseases.

## Material and methods

In this study, we performed extensive atomistic MD simulations of the CCR5 system in its apo form and also in the presence of ligands maraviroc and the chemokine CCL5, with two replicates (namely I and II). Additionally, we characterized the structural ensemble of CCR5 in complex with the experimental antagonists compound 21 (C21) and compound 34 (C34).

**Fig 1:**
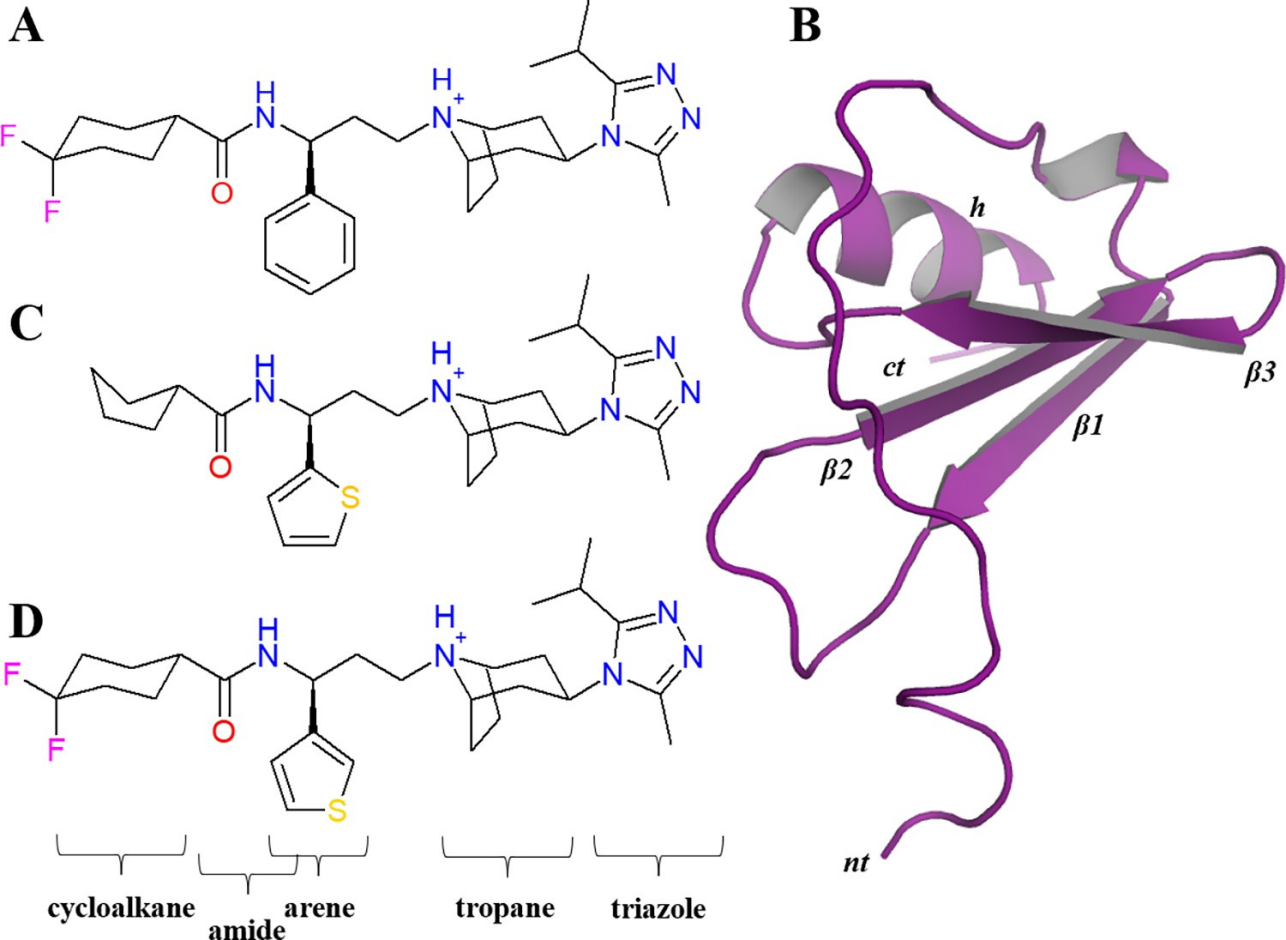
Structure of the ligands of CCR5. 2D representation of small-molecule ligands (A) maraviroc (MRV), (B) bounded chemokine CCL5[24–91]. The experimental ligands (C) Compound 21 (C21) and (D) Compound 34 (C34). Substructures of the ligands are indicated at the bottom of the figure. In CCL5 the segment at amine terminus is inserted in the part of the orthosteric site that overlaps with those of the small-molecule ligands. For the chemokine, *nt*: amino terminus, *ct*: carboxyl terminus, *β1–3*: beta sheets 1–3, *h*: helix.

The MD simulations were performed using, as initial coordinates, the experimental elucidated structure of CCR5 in complex with the partial inverse agonist [28] maraviroc (MRV) deposited in the Protein Data Bank (PDB ID: 4MBS [9]). For the agonist system, we based our CCR5-CCL5 model on the CCR5-[5P7]CCL5 complex (PDB: 5UIW [29] with the full CCL5[24–91] isoform (UniProt: P13501 [30]). We also took as initial models the ligand complexes of CCR5 with antagonists, bioactive molecules Compound 21 (C21) and Compound C34 (C34), that are also deposited in the PDB (PDB IDs: 6AKX and 6AKY respectively [31]). CCR5 tyrosine residues Y10 and Y14 were modeled as tyrosine-sulfate amino acids, according to experimental findings [32–34], and parameterized with ACPYPE [35]. All our models were membrane-oriented with the PPM server [36], and then embedded in a palmitoyl-oleoyl-phosphatidylcholine (POPC) lipid bilayer surrounded in water with NaCl 0.15 M using the *CHARMM-GUI* server [37, 38]. MD simulations were performed using *GROMACS* 5.0.4 package [39], with LINCS constraint algorithm for bonds involving hydrogen atoms [40], V-rescale thermostat at a reference temperature of 310 K [41], and semi-isotropic Parrinello-Rahman barostat with a reference pressure of 1 bar [42]. All simulations completed 600 ns, and the chemokine system was extended for 1 μs due to the temporal scale of activation of this system [43, 44]. Additionally, to assess the long-timescale of the functional changes of the GPCRs, and to compare our conventional MD (cMD) results, we performed Gaussian accelerated Molecular Dynamics (GaMD) sampling for the apo-receptor and CCL5-bounded complex systems. Initially, we performed a cMD using the SHAKE constraint algorithm for bonds containing hydrogens [45], a reference temperature of 310 K, and a Monte Carlo barostat with a reference pressure of 1.0 bar. Then, the GaMD sampling with dual boost on dihedral and total potential energy was performed in an NVT ensemble with upper limits of the standard deviation of the gaussian reweighting for both contributions of 6.0 kcal/mol was performed with *AMBER* 18 [46–49]. For the analysis of our simulations, we used ChExVis [50] to detect the interhelix water pore, in-home MDAnalysis scripts [51] to analyze the aqueous pore, R packages [52] to characterize the cross-correlation analysis of residues, and Bendix [53] to plot the helix bendings. PyMOL [54], VMD [55], and ISIS/Draw [56] software were used to make Figures and plots. Our CCR5 simulated systems are summarized in Table 1, and a 2D representation of their ligand structures is shown in Fig 1. The initial apo-CCR5 system was modeled from the CCR5-MRV structure without MRV.

**Table 1:** Simulated CCR5 systems and PDB IDs employed to build our initial structures.

System	CCR5 system details	Functional state	PDB ID	Simulated time (ns)	
				cMD	GaMD
CCR5	Apo	Basal	4MBS	600, 600	90
CCR5-MRV	Maraviroc	Inactivated	4MBS	600, 600	-
CCR5-CCL5	CCL5[24–91]	Agonized	5UIW, 6FGP	1000, 1000	150
CCR5-C21	Compound 21	Antagonized	6AKX	600	-
CCR5-C34	Compound 34	Antagonized	6AKY	600	-

## Results and discussion

### 1. Intracellular extremes of transmembrane helices 3, 5 and 6 are key regions to characterize the CCR5 functional state

**Fig 2:**
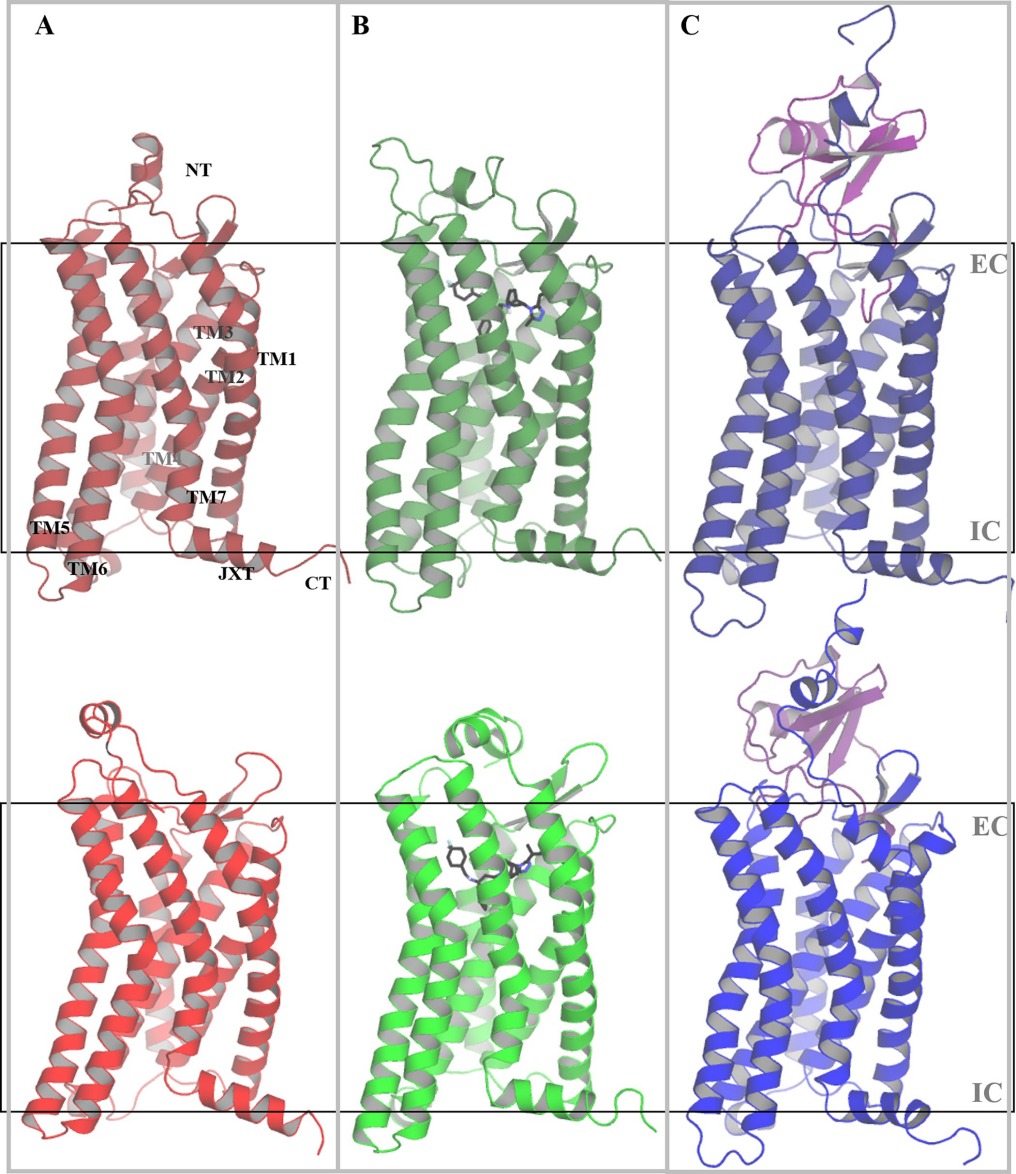
Representative structures of the systems of CCR5. (A) Apo-receptor-I (top) and II (bottom), and CCR5 in complex with (B) maraviroc-I and -II, and (C) chemokine CCL5-I and -II. Apo-CCR5 and CCR5-MRV adopt similar conformers. The chemokine in CCR5-CCL5 (in purple color) induces several structural changes quite distinctive to the MRV complex. EC: Extracellular side, IC: Intracellular side, TM1-6: Transmembrane helices 1 to 6, H8: Juxtamembrane helix (H8), NT: N-terminus, CT: C-terminus.

From our simulations, we were able to group our simulated systems into two categories based on their conformational similarities: (1) the *non-active state*, led by the partial inverse agonist ligand MRV (which presumably decreases the constitutive CCR5 activity [57, 58]) and the apo-receptor system, and (2) the *fully active state* that consists of CCR5 bound to the agonist CCL5 which is described to induce the activation of the receptor. Through a clustering analysis of transmembrane helices (TM), we found the most representative structures of each system (shown in Fig 2) and revealed some important differences among them:

**I.** The non-active state systems (apo-CCR5 and CCR5-MRV) showed a particular bending in TM5, which is very pronounced in CCR5-MRV (as found in a previous computational study of the mechanism of maraviroc [59]), and apo-CCR5 simulated systems. Also, a smaller tilt angle in TM6 for the simulated CCR5-MRV system (S1 and S2 Figs).
**II.** The *active* state system is constituted solely by our CCR5-CCL5 simulated system, mainly the chemokine interacting with the extracellular (EC) regions. The helicity of the intracellular (IC) segment of TM3 was extended in CCR5-CCL5 through the simulation time to the intracellular loop 2 (ICL2). The tilt angle in TM1 was increased, and the helicity of the IC extreme of TM5 and TM6 was decreased. We analyzed these findings by exploring the bending and tilt angles of the TM helices (S1 and S2 Figs).

### 2. The ligand interactions with a tetrad of residues of the orthosteric site are crucial for CCR5 activation

The CCR5 orthosteric site comprises an extensive region of CCR5 N-terminus (NT), extracellular loop 2 (ECL2), and a narrow hydrophobic cavity located at the extracellular side of the receptor where MRV binds, and also where the amino terminus (*nt*) from CCL5 first interacts to get inserted deep into the receptor interhelix region [28, 60]. Hereafter, the common part of the orthosteric site (OSS) consists of the hydrophobic cavity formed by a tetrad of residues: Y37^1.39^, W86^2.60^, Y251^6.51^, and E283^7.39^ (here, we use the Ballesteros-Weinstein nomenclature for the receptor residues [61]).

**Fig 3:**
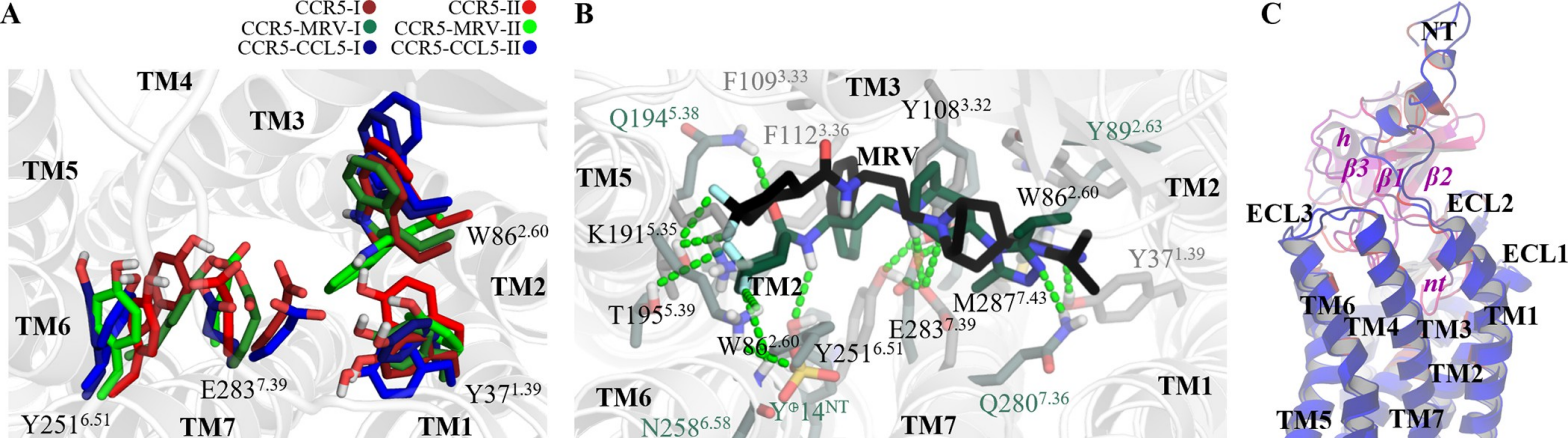
Orthosteric site of CCR5. Representative configuration of the OSS of the (A) three systems with replicates and complexes with (B) MRV and (C) CCL5. In the clustered configurations, the main interactions are the saline bridge between the tropane moiety with E283^7.39^ or Y108^3.32^, the ion-dipole E283^7.39^ with Y251^6.51^ or with Y108^3.32^, the hydrogen-bond between the triazole ring and Y37^1.39^, the aromatic interaction triazole-W86^2.60^, the interaction between at least one fluor atom and T195^5.39^, the aromatic interactions among the phenyl group of MRV and F109^3.33^ and F112^3.36^, and the hydrogen-bond between the ligand amide and Y251^6.51^. The interactions receptor-chemokine include the NT and all the TMs helices but TM4, as well as ECL2 and ECL3. The most variable contacts are those that are located within the NT due to its inherent higher mobility. The regions in CCL5 indicated in lowercase and italic text labels include the most extended peptide fragment except for helix (*h*). Despite its disordered segments, the unfolded residues of CCL5 do not exhibit greater motions than the NT of the receptor. The detailed contacts of CCL5 with CCR5 are presented in S3 Fig.

In our MD simulations, the apo-CCR5 system replicates show an expected configuration within the OSS since the basal state tends to adopt pro-inactive features. Thus, the CCR5-I and CCR5-II replicates resemble the CCR5-MRV-I system, particularly in the orientation and position of the tetrad (Fig 3). The CCR5-MRV-II replicate shows a different configuration within the OSS than the replicate I, although both, apo-CCR5 and CCR5-MRV, are congruent in an adjacent region to OSS, as we explain below. Also, the simulated receptor-ligand complexes stayed at the orthosteric site and conserved similar interactions to the crystallographic structures and published computational studies on CCR5 [59, 62–64]. Particularly, the interactions of the protonated tropane of MRV and E283^7.39^ and the hydrogen bond of the triazole with Y37^1.39^ are highly conserved.

Moreover, MRV interacts with T195^5.39^ through its fluorine atoms, in agreement with the experimental structure [9]. The triazole group stacks with W86^2.60^, whereas the indole sidechain points towards TM3. More detailed interactions of CCR5 with the ligands are in S3 and S4 Figs.

CCR5 and CCL5 interact through multiple residues and establish several types of interactions (S3 Fig). E283^7.39^ interacts with the backbone of the amino extreme of CCL5, and chemokine residues (referred to with lowercase code) *p*25, *y*26, and *s*27 interact with Y37^1.39^ and W86^2.60^ of the orthosteric tetrad, and Y89^2.63^. The deep location of residues *p*25 and *y*26 allows them to interact with M287^7.43^, which leads to local rearrangements in TM7 and influences the activation of CCR5, in agreement with previous experimental findings [34]. Y251^6.51^ showed high mobility and a small number of contacts with the chemokine, in contrast with MRV, these facts impact its interaction with the transmission switch W248^6.48^. The N-terminus region of the receptor forms predominantly ionic and ionic-backbone interactions with the chemokine residues; D2 and the two tyrosine sulfate residues Y10 and Y14 interact with lysine and arginine residues, *r*40, *k*68, and *r*70 from the chemokine. Several important residues in CCL5 that are post-translational modified, such as the *s27* and *s28* that can be glycated, *m90* that can be sulfoxidated [65], and the *s24*-*y26* tripeptide, that can be cleaved in many isoforms of CCL5, establish multiple contacts with CCR5. More details of the chemokine residues contacting CCL5 are also shown in S3 Fig. Sodium cations that interact with the apo-receptor but not with MRV nor CCL5 complexes are reported in S5 Fig.

**Fig 4:**
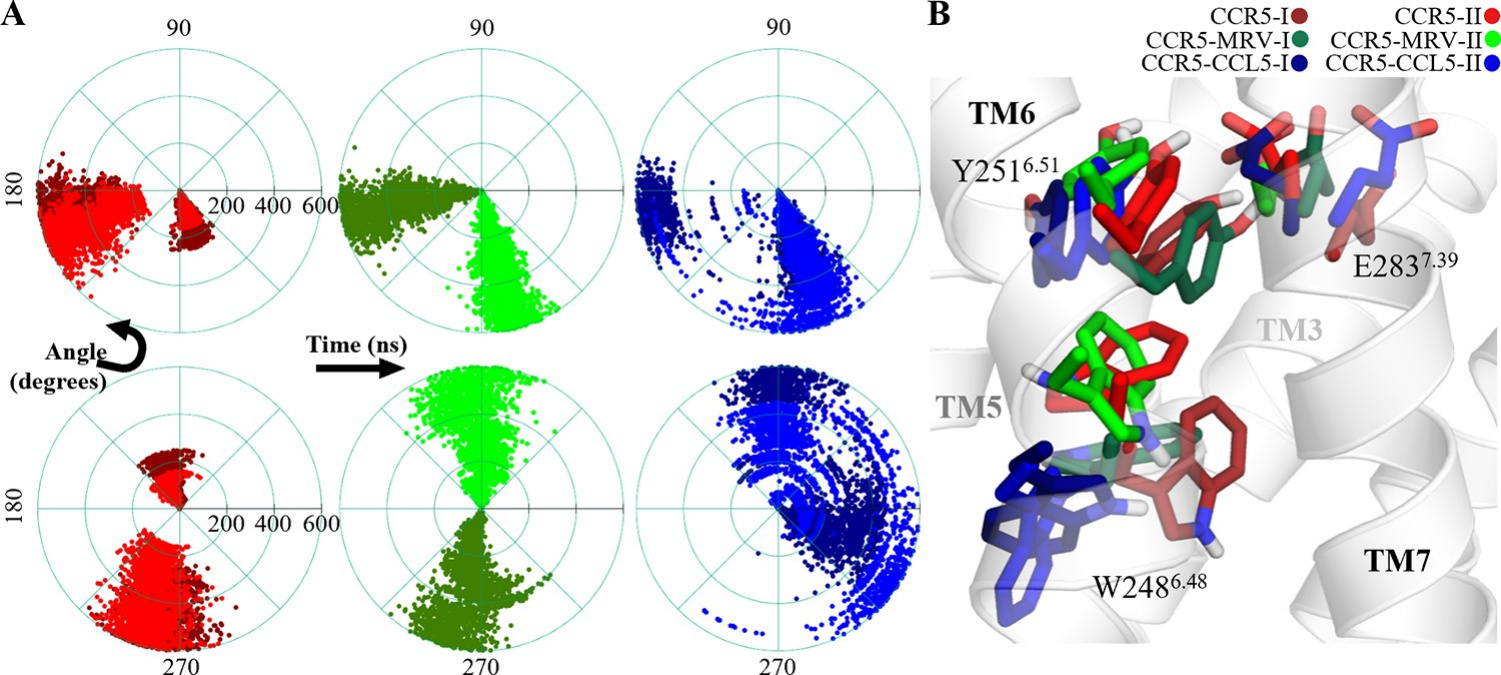
The link between the orthosteric site and the transmission switch. (A) Angular plots of the rotamers of W248^6.48^, and (B) superposed configurations and the pair Y251^6.51^-E283^7.39^, where W248^6.48^ is oriented towards TM5 only in the chemokine complex. The angular plots are for χ_1_ (top) and χ_2_ (bottom) dihedrals of W248^6.48^.

Interestingly, residue Y251^6.51^, which is part of the OSS, interacts with the adjacent *transmission switch*. The transmission switch is composed of conserved residues that link the binding site with the movement of the TM helices that activate the receptor. In CCR5, this transmission switch is formed by W248^6.48^, and is located at the conserved WXPY motif [9, 34, 58] and has been previously used as an indicator of the functional state of Class A GPCRs [43, 66]. The switching amino acid couples the orthosteric site, through Y251^6.51^, and the *aromatic connector* [34], that is the hydrophobic region formed by Y108^3.32^, F109^3.33^, and F112^3.36^, immersed in turn in the *hydrophobic layer 1* (HL1) [67], which is a region that in CCR5 contains four phenylalanine and one tyrosine residue. The distances between Y251^6.51^ and Y108^3.32^ from E283^7.39^ are indicators of activation switching since the greater distance among them allows the coupling between the orthosteric site and the transmission switch (S6 Fig), and thus, W248^6.48^ positions its indole ring either close to TM5 or towards the receptor pore. In the apo-CCR5 and CCR5-MRV systems, the configuration of Y251^6.51^ and E283^7.39^ are equivalent, with a displacement of TM7 in the replicate I of the complex, with respect to replicate II of the apo-system (Fig 4).

In the CCR5-CCL5 system, Y251^6.51^ access rotamers that direct its sidechain to the W248^6.48^ vicinity and the *PIF cluster* (P206^2.50^, I116^3.40^, and Y244^6.44^), diminishing the interaction with E283^7.39^ and the ligand. The χ_1_ and χ_2_ dihedrals of W248^6.48^, which are formed by the atoms N-Cα-Cβ-Cγ and Cα-Cβ-Cγ-Cδ1, respectively, show distinctive values that indicate the torsion related to the activation of this transmission switch (Fig 4). The activation of the transmission switch collapses HL1 and determines the remodeling of the interhelix pore. In our extended simulation of 1 μs and in the GaMD simulations of CCR5-CCL5, the torsion W248^6.48^ is consistent with the observed in the 600 ns of cMD simulation. In the prior, the torsion is maintained until the last, and for the latter, the torsion is reached from the beginning (S7 Fig), confirming that the orientation of this residue is a conformational hallmark of the activation of CCR5, according to the reported for other GPCRs of Class A. The GaMD simulation of apo-CCR5 also shows the initial torsion of the transmission switch in the first 230 ns of the cMD simulation, suggesting that the latter may tend to a non-active functional state that in the prior is not accessible.

### 3. Water penetration of the whole interhelix pore characterizes the activating state of CCR5

**Fig 5:**
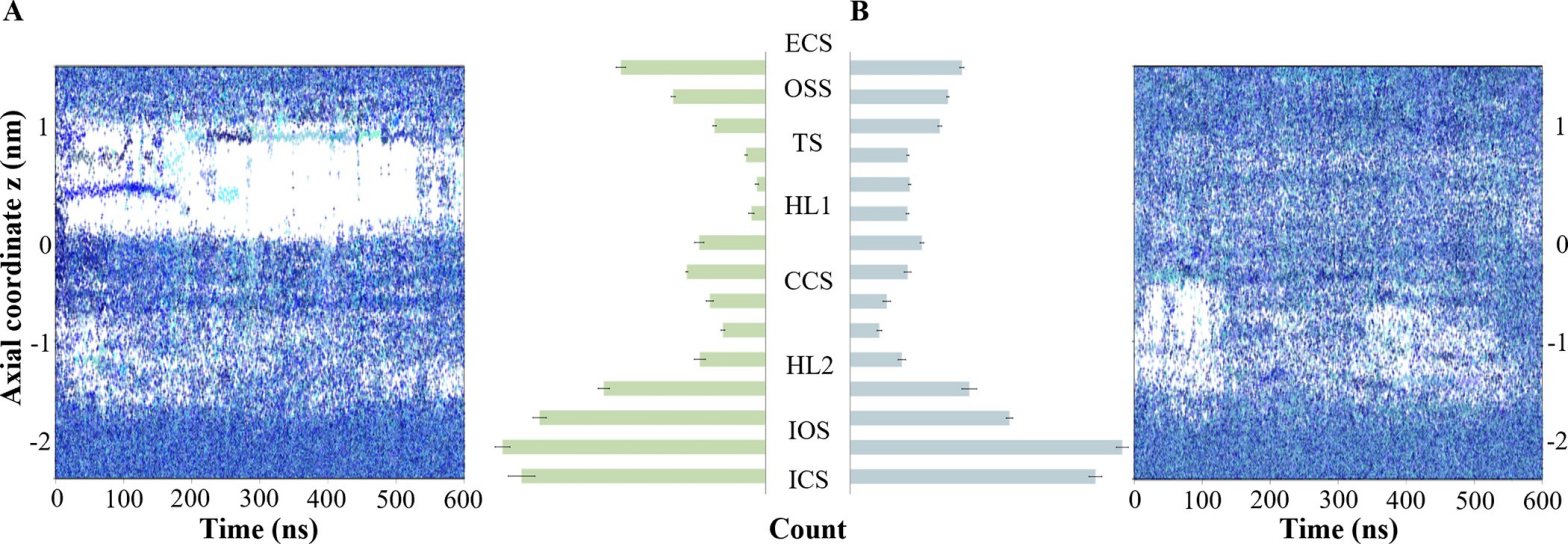
Water molecules are present in the interhelix pore through the axial coordinate. (A) the CCR5-MRV, and (B) CCR5-CCL5 systems. In the center, the barplot represents the average and standard error of the mean of the number of water molecules over intervals of 100 ns from the equilibrated structure to the 600 ns. Each bar comprises the partition time and segments for an average of 0.5 nm through the axial coordinate. For the inactive system, the region ranging the orthosteric site (OSS), the transmission switch (TS) is predominantly dehydrated, with the hydrophobic layer 1 well conformed, whereas the CCS and the ionic lock switch (IOS) is hydrated. In the agonized system, the presence of water molecules is extended through all the pore beyond the 140 and until the 600 ns, without integration of HL1. The histograms also show the collapse of the HL1 in the inactive system, as well a high abundance of water within the pore.

The collapse of HL1 in CCR5-CCL5 system leads to water entrance from the OSS through the interhelix pore and hydrating the path to the central coordination site (CCS) at the middle, where a conserved residue D76^2.50^ is present. In contrast, the CCR5-MRV water presence pattern remains dehydrated in a wide region in HL1 (Fig 5). We calculated the number of water molecules in the pore through axial segments of 0.5 nm every 100 ns for these two systems. Counting the water molecules within the heptahelical bundle, the inactive system exhibits less overall water presence in the pore, moreover HL1. In the continuous path of hydration in the chemokine system, the hydrophobic layer 2 (HL2) [67] constitute a bottleneck that is very straight at the beginning of the simulation and then relaxes. HL2 is comprised of several hydrophobic residues such as L69^2.43^, L72^2.46^, I119^3.43^, L122^3.46^, M210^5.54^, Y214^5.58^, L236^6.36^, I237^6.37^, I240^6.40^, and Y297^7.53^. These residues are part of two functional regions. The pair L236^6.36^ and I237^6.37^ represents a hydrophobic switch whose configuration determines the features of the bottleneck. (S8–S10 Figs). When the sidechains of these residues due to a rotation of TM7, as principal component analysis (PCA) showed (S11 Fig), HL2 collapses, and then the water molecules penetrate from CCS to the ionic lock site, the adjacent region formed by Y214^5.58^ and Y297^7.53^, part of HL2; R126^3.50^ and R235^6.35^.

The pentad of D125^3.49^, R126^3.50^, Y214^5.58^, R235^6.35^, and Y297^7.53^ form several paired interactions, some of them related to the TM6 rotation and water presence in the pore (S12 Fig). The ionic interaction between R232^6.32^ and E302 predominates in CCR5-CCL5, whilst in apo-CCR5, it is infrequent. The rotamers that lay the sidechain of L236^6.36^ towards the pore are frequent in the apo-system, whilst those with MRV and CCL5 clear the pore of the sidechain. These interactions suggest the reordering of the IC region of TM3 and TM5-TM7. The water presence in the IC part of the pore seems to influence most of these interactions and motions.

### 4. The activation mechanism of CCR5 involves Y251^6.51^ pushing onto W248^6.48^, TM5 and TM6 tilting outward, and the modification of the ionic lock as the information propagation path from the orthosteric site to internal CCR5 regions

The cross-correlation analysis of the CCR5-CCL5 α-carbons dynamics shows that a cluster of residues in TM1, TM2, and juxtamembrane helix (H8) forms a community highly correlated with the CCS and indicates the concerted motion of TM1 and TM2 by the H8 gyration shown in S11 Fig. The negative cross-correlation values include three clusters of residues: *a)* The subdomain from CCS to EC through TM1-3, including Y108^3.32^ and the kink at TM1; *b)* the EC part of TM6 with H8. And *c*) two subdomains of the IC segments of TM5 with TM6 (S13 Fig). These findings show a concerted motion between the surrounding of Y108^3.32^ and D76^2.50^ and thus, the orthosteric site and the CCS; the opening of the pore by the movement outward of the EC part of TM6 with the downward bend of H8, and the scissoring motions of the IC parts of TM5 and TM6, which were also evident in our PCA analysis.

**Fig 6:**
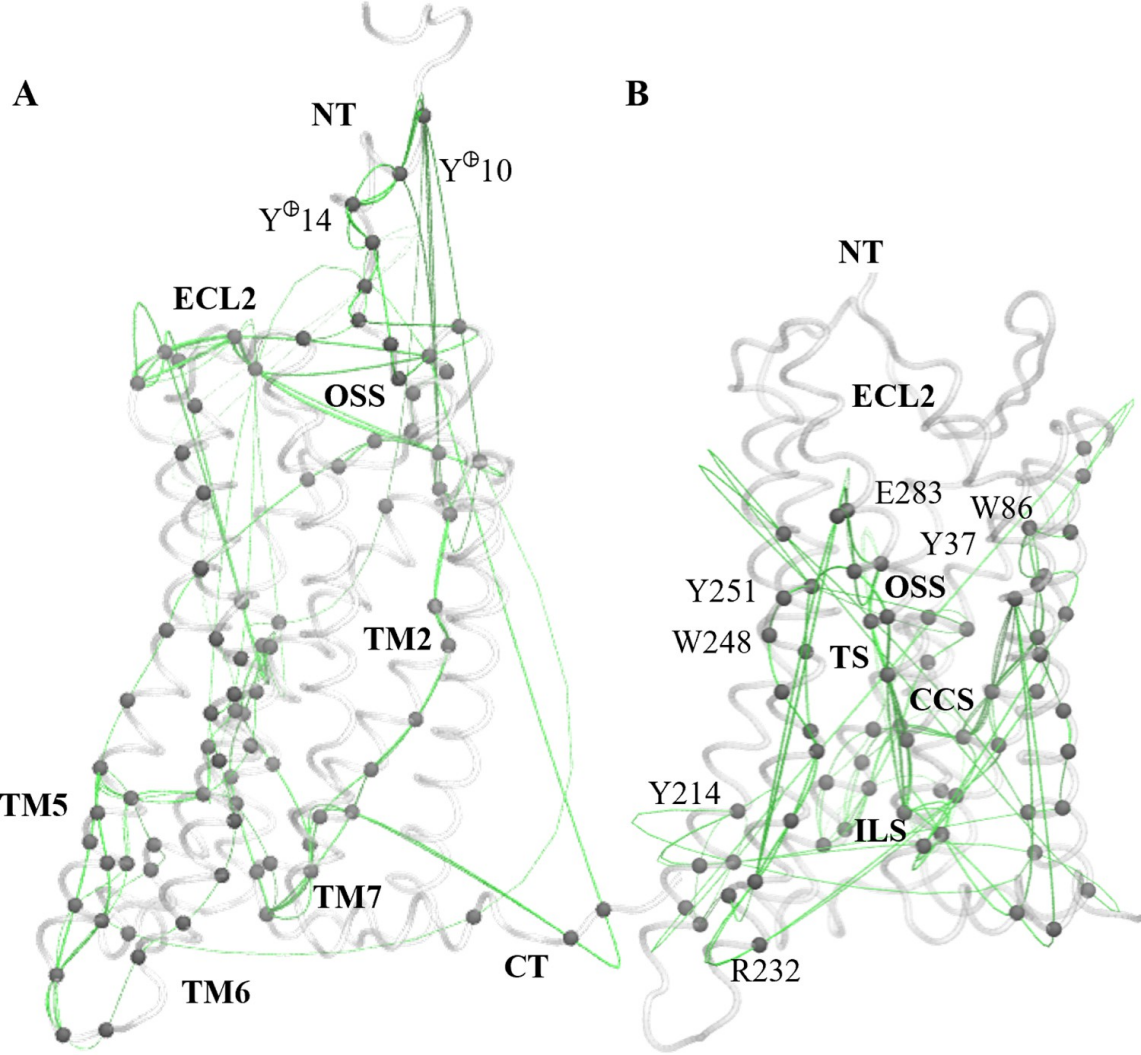
Cross-correlation network analysis of the CCR5-CCL5 system shows similar propagation paths of correlated motions. We looked for pathways of correlated amino acid motions from the EC side to the IC side of the receptor. (A) Paths involving the N-terminus and EC side of TMD to the IC region. Correlated motion pathways pass through TM2, TM5, and TM6 predominantly, and are not included in many of the key sites. (B) The paths from the orthosteric site (OSS) to the ionic lock site (ILS) span an extensive part of the TMD and include notable residues such as Y37^1.39^, W86^2.60^, Y251^6.51^, and E283^7.39^ from OSS; W248^6.48^ from the transmission switch (TS), part of HL1, D76^2.50^ in the central coordination site (CCS), and Y214^5.58^, R232^6.32^, L236^6.36^ and Y297^7.53^ within the ionic lock site (ILS). Other path analyses are illustrated in S15 Fig.

The community network analysis among the five different systems also suggests the multiple differences in the receptor dynamics. The larger communities suggest extensive concerted motions, such as CCR5-MRV, that are consistent with the aqueous pore features. In contrast, the fragmented communities along the receptor may be related to the water dynamics through the pore (S14 Fig). Since apo- and MRV systems have lesser water within the pore, the ligand absence and the Na^+^ coordination may be responsible for the multiple communities in that system. We found that CCR5-MRV has communities that involve many residues, distinct from the rest of the systems. Nevertheless, the cross-correlation networks respond to multiple variables and not only to the water presence. We analyzed the shortest paths of propagation between different subdomains in CCR5 through two approaches: the entire backbone and the TM domain (TMD) with the ECLs and ICLs. Focusing on the EC and the IC subdomains as extremes, we found that the N-terminus participates in the paths, including the two tyrosine sulfate residues, the EC part of TM1, ECL2 (including the orthosteric site), and passes across TM2, TM5, TM6, and H8, spanning several important regions but not some key residues such as D76^2.50^, E283^7.39^, W248^6.48^, or Y297^7.53^. Nevertheless, analyzing the TMD backbone, the orthosteric site, and the ionic lock-associated residues as extremes, the paths include all the residues that we examined above, *i*.*e*., those from the orthosteric site, transmission switch, HL1, central coordination site, and ionic lock site (Fig 6). In the TMD network analysis, TM1-3, TM6-7, and H8 are important for the shortest propagation paths; and to a lesser extent, TM5. To inquire about the connection between multiple regions, we also searched for communication paths between subzones in the TMD that revealed several networks among the different switches. These paths connect different key regions, such as the orthosteric site with the transmission switch and the ionic lock site, the central coordination site, and the ionic pair R232^6.32^-E302^H8^ (S15 Fig).

Integrating all the findings, we found that the partial inverse agonistic mechanism of MRV involves primarily the Y251^6.51^-E283^7.39^ interaction and the W248^6.48^ rotamer directed to the pore or TM7, as well as the HL1 and its dehydrated surrounding. The barrel-shaped adopted by the TMD allows longer distances and certain rotamers within some residues at the ionic lock site. Our apo-CCR5 system shares many features with the inactive system, but some features are distinguishable, such as the dehydration of HL2 and the interaction with sodium cations, absent in the other systems. For the chemokine system, we claim that the activation mechanism of CCR5 with the bound chemokine CCL5 in our system involves a large network of residues located in the orthosteric site, through Y251^6.51^ and W248^6.48^ as a communication pathway, both disrupting and collapsing HL1 and allowing the water molecule patterns into the interhelix pore. This differential hallmark in the CCR5-CCL5 activating system influences the reordering of the receptor IC, with motions such as the gyration of helix segments, scissoring, and bending of helix extremes. And at the amino acid level, we found torsions and interactions in key residues that, altogether with the rest of the mentioned conformational events, result in the conformational ensemble of an activated receptor uncoupled from the G protein complex. Nevertheless, our multiple activation-related findings agree with the reported features from active ensembles in class A GPCRs. Additionally to this concordance, we compared the experimental structure of CCR5 with a modified version of CCL5, the super-agonist [6P4]CCL5, deposited in the Protein Data Bank once we started with this study (PDB ID: 7O7F [34]) to identify similarities. From these analyses, we found that in our extended simulation of CCR5-CCL5, a decrease in the backbone RMSD of our simulated system with respect to the experimental structure 7O7F, from 0.33 to 0.15 nm (S16 Fig), suggesting a feasible convergence of our conformational ensemble with the experimentally determined fully-activated configuration, despite the absence of the G protein coupling.

### 5. CCR5 structural features induced by the binding of a partial inverse agonist, maraviroc, are distinctive to those led by the binding of antagonists compound 21 and compound 34

MD simulations that started from the experimentally determined structures of CCR5 bonded to antagonists C21, and C34 sampled similar structural ensembles to the apo-receptor. From the clustering analysis mentioned in the first section of the study and shown in Fig 2, the receptor in the presence of C21 and C34 exhibited similar conformations of TM5 and TM6 to the ones sampled by the apo-CCR5 simulations. The general structure of CCR5-C21 and -C34 did not show a barrel-shaped geometry (S17 Fig). Nevertheless, the orientation of the transmission switch W248^6.48^ for the C21 complex resembles CCR5-MRV-II, whilst the transmission switch orientation in the C34 complex agrees with the CCR5-MRV-I replicate, both as non-active states (S18 Fig). Analyzing the interactions of these bioactive antagonists, we found similar patterns to the CCR5-MRV ensemble, such as the torsion of W86^2.60^ and the hydrogen bond between Y37^1.39^ and the triazole of the small-molecule ligands. Nevertheless, C34 presented a subtle displacement within the orthosteric tetrad that led to a disruption of the ionic bridge between E283^7.39^ and the protonated tropane in favor of the Y108^3.32^ interaction (S4 and S19 Figs). C21 and C34 complexes presented a dehydrated interhelix pore, similar to the non-active systems (S8–S10 Figs).

## Conclusions

Using extensive atomistic MD simulations, we identified key CCR5 conformational changes induced by the binding of a partial inverse agonist that are quite distinctive to those led by the presence of CCR5 antagonists. Although there are some similarities, we consider that these changes may constitute another functional state in CCR5 within the spectrum of functional conformational states known for GPCRs. Thus, in the presence of the partial inverse agonist MRV, the receptor reaches an inactive state with the constitutive activity diminished. Whereas our MD simulations of the CCR5 receptor in complex with C21 and C34 constitute antagonized states, different from the inactive state that we refer to as the *stative* states, due to the known blockade of the antagonists in the functional state change and the binding of agonists.

## Supporting information

S1 Fig Curvature of the TM helices of the five systems of CCR5, in degrees through the simulation time. Despite the many differences between CCR5 and CCR5-MRV with CCR5-C21, the bending of their helices is very similar. In contrast, the systems CCR5-C34 and CCR5-CCL5 display a TM3 extension that imply the high curvature of the helix to continue with the ICL2.
(TIF)

S2 Fig Tilt angles of the helices. The selection of the segments to the calculation of the tilts were based on the helicity of the residues, mostly at the kinks, through the simulation time. The circular plots display the extracellular (EC) and intracellular (IC) portions of the helices, except TM3 and H8. The most notable differences are: TM1-EC between MRV and CCL5, TM1-IC between C21 and CCL5, TM2-IC between MRV and CCL5, TM2-EC between C34 and CCL5, TM3 between MRV and C34, TM4-IC between C21 and CCL5, TM4-EC between MRV and CCL5, TM5-EC between C34 and CCL5, TM5-IC between C34 and apo, TM6-IC between apo and CCL5, TM6-EC between MRV and CCL5, TM7-EC between apo and CCL5, TM7-IC between C21 and CCL5, and H8 among apo and MRV, C21 and C34. The relevant findings are the greater difference in inclination between the inactive system CCR5-MRV, with the chemokine complex CCR5-CCL5, maximal in TM4, TM5, TM6 and TM7; the difference in apo-CCR5 in TM5m TM6-IC and TM7; and the multiple differences among the complexes of MRV, C21 and C34.
(TIF)

S3 Fig Contacts in the receptor-ligand complexes. (A) Barplot of the scaled distances lesser than 4.5 Å, among the three small-molecules MRV (replicates I and II), C21 and C34 with each residue in CCR5. (B) Plot of the proportion of the distance value for the amide, tropane, and triazole moieties in the small-molecule ligands, with respect the distance in the experimental structures. (C) Interactions of CCL5 in the CCR5 representative conformer of the replicates I and II. The chemokine residues are in lowercase. (D) Plot of the interaction types in the CCR5-CCL5 complex. The inner ring counts for the hydrophobic and the outer for the polar interactions between the receptor and the chemokine. Each color represents the type of interaction, and the relative size of the circles indicates the closeness of the CCR5 residue to CCL5.
(TIF)

S4 Fig Interaction between the ligand and the aromatic residues of the hydrophobic layer 1 (HL1). (A) MRV, (B) C21 and (C) C34. The three ligands are positioned with the arene ring towards HL1, and for MRV and C21, surrounded by it, particularly F109 and F113 at the TM3 side, W248 and Y251 at TM6 side, and F112 at a hydrophobic pocket among TM3, TM5 and TM6. For C34, the thienyl ring are displaced from the hydrophobic environment, and exposed to the interhelix side of TM5 and TM6. (D) Clustered barplots, grouped by small-molecule ligand systems, indicating the proportion of higher or lower distance from each residue in CCR5 to the arene moiety, with the distance from experimental structures as reference. MRV phenyl ring only exhibits large distancing from I198, whilst comes near to the rest of the residues. C21 shows distancing from all the residues excepting Y251, compared with the other two ligands. C34 establishes contact with several residues that MRV and C21 do not, such as T195, V199, L203, N252, L255, and M279, being the two later the closer residues than in the experimental complex. Some residues are in contact with the small-molecule ligands only in one or two systems, and in a larger distance in all of these cases.
(TIF)

S5 Fig Coordination of Na^+^ with E283^7.39^ of the orthosteric site. (A) apo-CCR5 (replicate I), and (B) CCR5-C34 systems. D276 and S272 coordinate a Na^+^ in apo-CCR5 system. Tyrosine sulfate residue (Y14) are not available to coordinate sodium cations since it interacts with either (A) R168 or (B) Y187 backbone. (C) Minimal distance of E283 carboxylate to any Na^+^.
(TIF)

S6 Fig Minimal distance between E283 of the orthosteric site. Distances from (A) Y251 and (B) Y108. In CCR5-CCL5 system, Y251 moves away from E283 to W248 vicinity in a greater extent than the other systems, and lesser in CCR5-MRV and CCR5-C21. Y108 remains closer to E283 in CCR5-C21 and apoCCR5, whilst it displaces farther in CCR5-CCL5 and CCR5-C34, suggesting that the chemokine, solely structural difference is not responsible of the separation of that pair.
(TIF)

S7 Fig Torsion angles χ_1_ and χ_2_ of W248^6.48^ for the apoCCR5 and CCR5-CCL5 systems. (A) From the 1 μs extended simulation of CCR5-CCL5-I, where the torsion reached in the short version of 600 ns keeps. (B) From the GaMD systems for apoCCR5 and CCR5-CCL5 systems. Both torsion angles for apo-receptor are consistent with the cMD simulation before the change at 230 ns, suggesting the lack of a change of state in the transmission switch. For chemokine-bound receptor, the χ_1_ angle reaches the same configuration of the cMD at 440 ns, and the χ_1_ angle assumes a different value, but (C) a configuration of the indole sidechain oriented to TM5 and water pore as in the cMD system of CCR5-CCL5-II. In the apo-receptor system the configuration is quite similar than the cMD simulation.
(TIF)

S8 Fig Water presence in the interhelix pore. (A) apo-I and II, and small-molecule ligand systems (B) C21, (C) MRV-II and -II, (D) C34, and (E) CCL5-I and -II. HL1 and CCS regions are similar in the four systems, with a slightly more hydrated pattern in CCR5-C34. The apo-CCR5 system shows a narrow, constant dehydrated region in HL2, whilst the MRV, C21 and C34 present variable patterns in this region. C21 system was more hydrated at HL2, and C34 complex displays a broad dehydrated region between CCS and HL2 for about 300 ns of the simulation.
(TIF)

S9 Fig Water presence in the interhelix pore in the GaMD simulations. (A) apo-CCR5 and (B) CCR5-CCL5 systems. In the prior the dehydrated zone remains and, in the latter, a hydrated pattern like the cMD simulation is reached since the beginning.
(TIF)

S10 Fig Accumulated water presence in each system. For the cMD simulations: (A) apo-CCR5-I and -II, (B) CCR5-MRV-I and -II, (C) CCR5-C21, (D) CCR5-C34, and (E) CCR5-CCL5-I and -II. For the GaMD simulations: (F) apo-CCR5 and (G) CCR5-CCL5 systems. In the non-active systems is evident the interruption of the water continuous path in the HL1 mainly, and in a lesser extent in HL2, but in the accelerated simulation. In both sampling schemes, the chemokine-bound systems possess a continuous water path overall the interhelix pore.
(TIF)

S11 Fig Principal modes of TMs in CCR5-CCL5-I system. The motion begins in the reddish and gray colors and ends in blue color. (A) Principal mode 1 (PC1) of TMD, representing the % of the total essential variance of motion. (B) PC1 within TM5, TM6 and TM7 helices, that shows the distinctive motions between the EC and the IC part of each helix. The EC part of TM5 make a left-handed gyration, whilst the IC part makes a displacement towards TM6. The EC part of TM6 moves away the TMD and the EC region, and the IC segment makes a scissoring bend to TM7 and to the upper direction. The IC part of TM7 displaces to TM6 with a torsion. (C) H8 and its adjacent TM7 portion make an anti-symmetrical stretching, whereas H8 moves to TM1, displacing it. (D) The PC2 of TM7shows a larger motion of their IC part to the inter-helix pore. (E) The global helix motions in PC1 of TM7 shows the anti-symmetrical motion with H8, the prior towards the pore and the later to TM1, where (F) it is displaced by H8 to TM2. (G) The gyration of the IC part of TM7 is right-handed, as is evident with the porcupine representation of the backbone. (H) The sidechains of the same region gyrate like the backbone, excepting Y297, whose sidechain moves to the other direction. Thus, this contrary motion of Y297 let the sidechain remaining to the pore.
(TIF)

S12 Fig Ionic lock-associated residues. D125^3.49^, R126^3.50^, Y214^5.58^, R235^6.35^, Y297^7.53^, and L236^6.36^, for (A) apo-CCR5, (B) CCR5-MRV, (C) CCR5-C21, (D) CCR5-C34, (E) CCR5-CCL5, and (F) all systems. The quadrilateral labels in gray color represent the average distance among the Cα atoms at TM3, TM5, TM6 and TM7 respectively. The notable features are the long distances between 1: R126 and Y214 in CCR5-MRV; 2: Y214 and R235 in CCR5-MRV; 3: R235 and Y297 in apo-CCR5, CCR5-C21 and CCR5-CCL5; and 4: Y297 and R126 in CCR5-C21. The rotamers that lay the sidechain of L236 towards the pore are frequent in the apo- system, and in the complexes with C21 and C34, whilst in those with MRV and CCL5 clear the pore of the sidechain.
(TIF)

S13 Fig Pearson cross-correlation along time of carbon alpha of TMs in CCR5-CCL5-I system. The significative residue network in TMs is for (A) positive cross-correlation within 0.6 to 0.8, and (B) negative cross-correlation within -0.6 to -0.8. The positive cross-correlation in the concerted motion is centered in TM1, TM2 and H8, from the CCS to the IC zone, related with the principal mode 1 in this region. The negative cross-correlation motions involve residues of TM1, TM2 and TM3, for CCS to the EC side. Also, two residues in in TM6-EC and one in TM1-IC, suggesting the opening of the pore and the acquisition of a trapezoidal form of the receptor. Between TM5 and TM6, at the IC side, two paired zones are correlated, involving Y127 of the DRY motif, and the distal part of both helices in the IC face, suggesting a scissoring bend between TM5 and TM6.
(TIF)

S14 Fig Community network analysis over the TMD region of the CCR5 systems. Each community represents a large, adjacent Pearson cross-correlation among the Cα atoms for (A) apo-CCR5, and (B) CCR5-MRV system; (C) CCR5-C21, (D) CCR5-C34, and (E) CCR5-CCL5 systems. The color of each sphere indicates the high correlation within (in descendent order: blue, red, dark gray, orange, yellow, light gray, green, white, pink, cyan). The relative size is related with the extension and ponderation of the positions of every correlated member in the community. It is noticeable that the CCR5-MRV and CCR5-C34 systems show extensive communities (first and second respectively) through the TMD region. In contrast, the apo-CCR5, CCR5-C21 and CCR5-CCL5 exhibit very partitioned patterns overall the communities, suggesting a perturbation that may be related with the water dynamics within the interhelix pore.
(TIF)

S15 Fig Four selected paths of the cross-correlation propagation in the Cα atoms of the backbone of CCR5. (A) Orthosteric site and transmission switch, (B) orthosteric site and ionic lock region, (C) transmission switch and central coordination site, and (D) transmission switch and R232-E302 ionic pair.
(TIF)

S16 Fig Comparison of our CCR5-CCL5-I and -II systems with the experimental structure (PDB ID: 7O7F), a complex of CCR5 with a modified CCL5 chemokine, the super-agonist [6P4]CCL5. (A) The RMSD of the backbone with a defined secondary structure (*i*.*e*., helices and beta-sheets) were calculated on our trajectory with respect 7O7F. The predominant decrease in the RMSD of replicate I suggests that our system approximates to the full activated state, despite the bias introduced by the CCL5 mutations in 7O7F. (B) Superposition of both complexes, our simulated system (blue color) and the experimental system (light gray color).
(TIF)

S17 Fig Representative conformers of the five systems from the clustering analysis. (A) apo-CCR5, (B) CCR5-MRV, (C) CCR5-C21, (D) CCR5-C34 and (E) CCR5-CCL5. The main differences are the barrel-shaped geometry of the inactive conformer and the separation of the IC extremes of TM6 and TM7.
(TIF)

S18 Fig Torsions of W248^6.48^ of the five systems. (A) apo-system, and complexes with MRV, C21, C34 and CCL5. (B) Configuration of the sidechain of W2486.48 in the backbone-aligned clustered structures.
(TIF)

S19 Fig Small-molecule ligands bounded with CCR5. (A) MRV, (B) C21 and (C) C34 in the cavity of the orthosteric site.
(TIF)
