# Supplementary figures and images for: Computational study of the structural ensemble of CC chemokine receptor type 5 (CCR5) and its interactions with different ligands
Source: PLoS One. 2022 Oct 17;17(10):e0275269. doi: 10.1371/journal.pone.0275269 (PMC9576088; doi:10.1371/journal.pone.0275269)

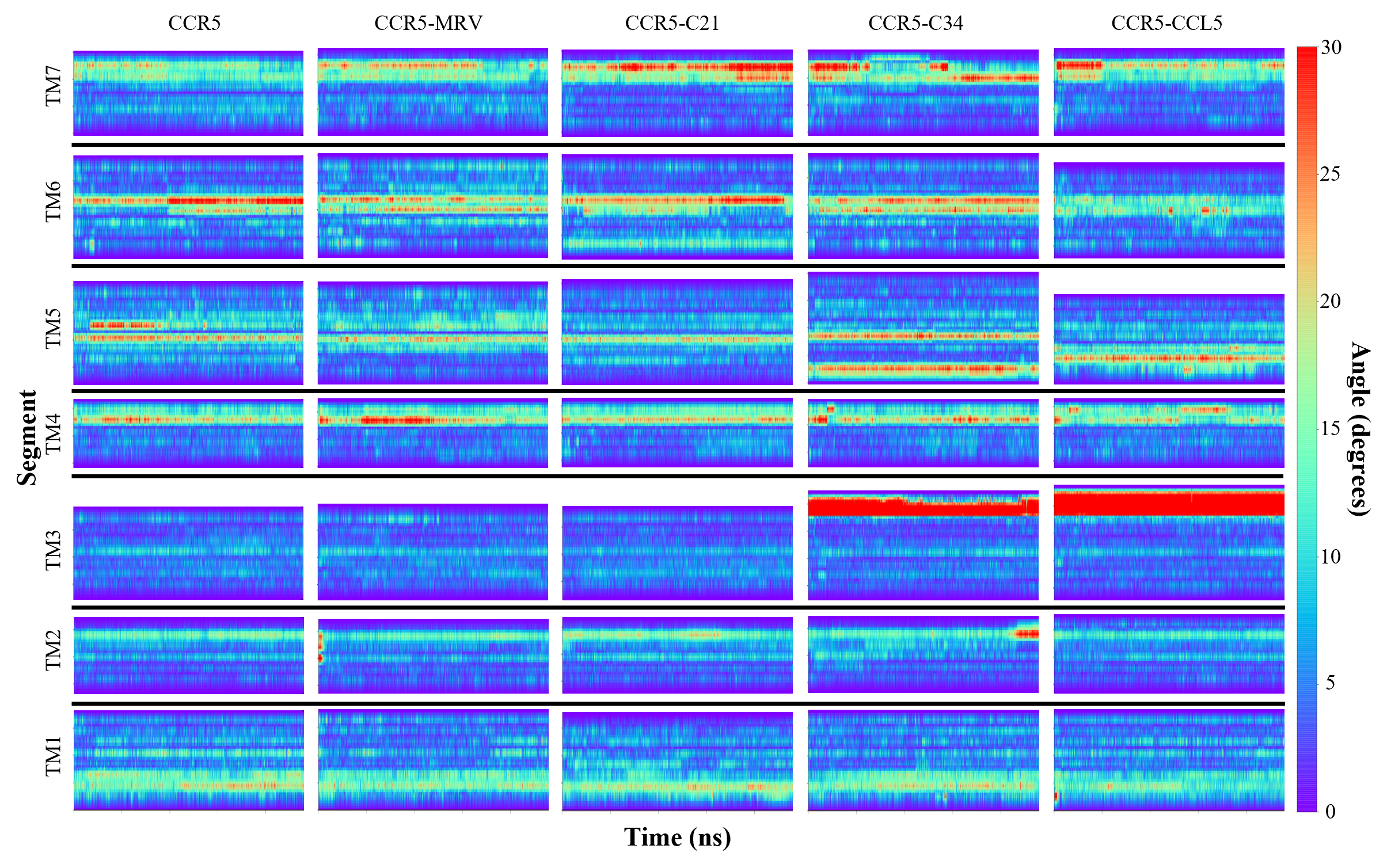

Supplement: S1 Fig — Despite the many differences between CCR5 and CCR5-MRV with CCR5-C21, the bending of their helices is very similar. In contrast, the systems CCR5-C34 and CCR5-CCL5 display a TM3 extension that imply the high curvature of the helix to continue with the ICL2. (TIF) [file pone.0275269.s001.tif]

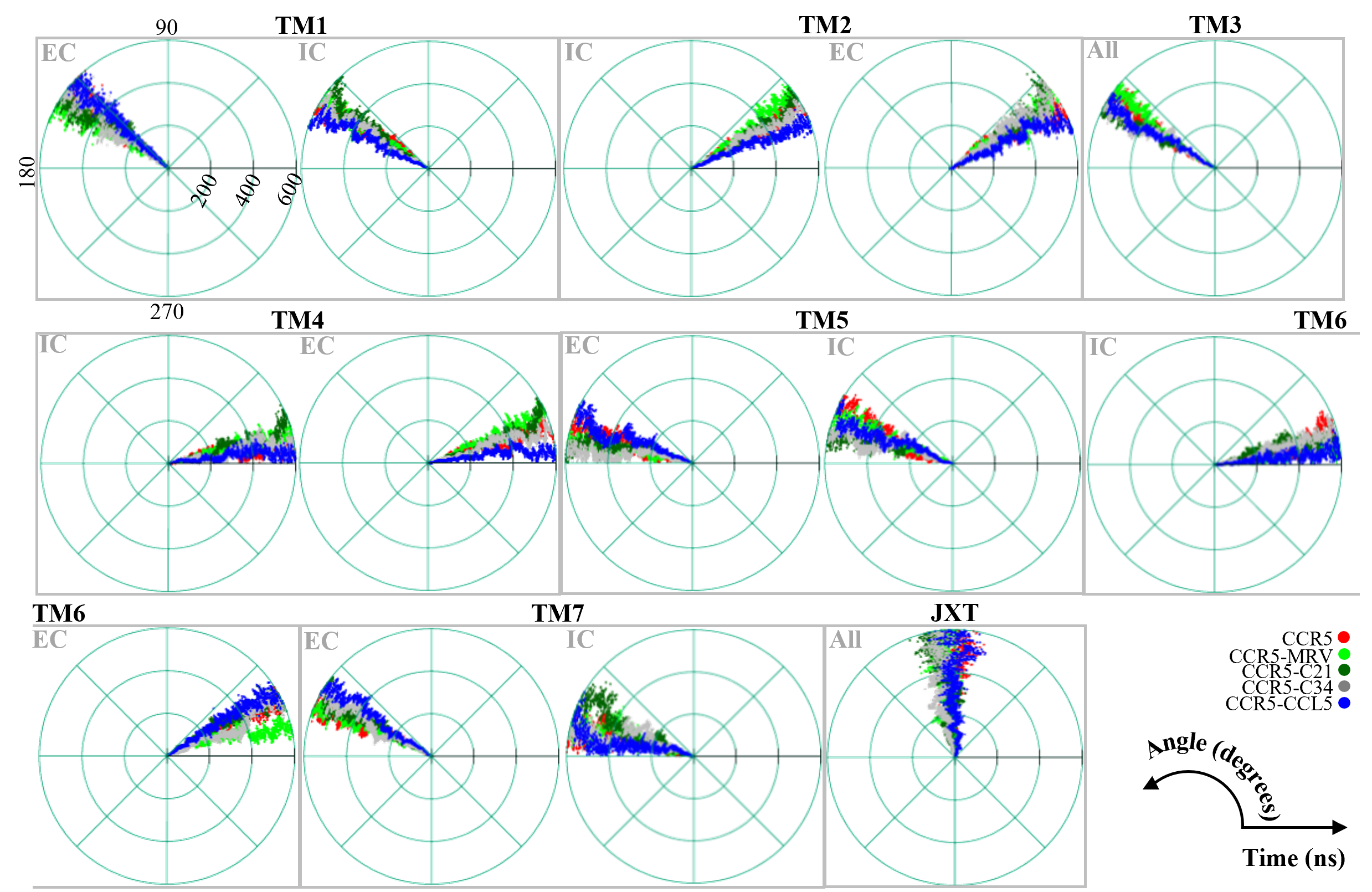

Supplement: S2 Fig — The selection of the segments to the calculation of the tilts were based on the helicity of the residues, mostly at the kinks, through the simulation time. The circular plots display the extracellular (EC) and intracellular (IC) portions of the helices, except TM3 and H8. The most notable differences are: TM1-EC between MRV and CCL5, TM1-IC between C21 and CCL5, TM2-IC between MRV and CCL5, TM2-EC between C34 and CCL5, TM3 between MRV and C34, TM4-IC between C21 and CCL5, TM4-EC between MRV and CCL5, TM5-EC between C34 and CCL5, TM5-IC between C34 and apo, TM6-IC between apo and CCL5, TM6-EC between MRV and CCL5, TM7-EC between apo and CCL5, TM7-IC between C21 and CCL5, and H8 among apo and MRV, C21 and C34. The relevant findings are the greater difference in inclination between the inactive system CCR5-MRV, with the chemokine complex CCR5-CCL5, maximal in TM4, TM5, TM6 and TM7; the difference in apo-CCR5 in TM5m TM6-IC and TM7; and the multiple differences among the complexes of MRV, C21 and C34. (TIF) [file pone.0275269.s002.tif]

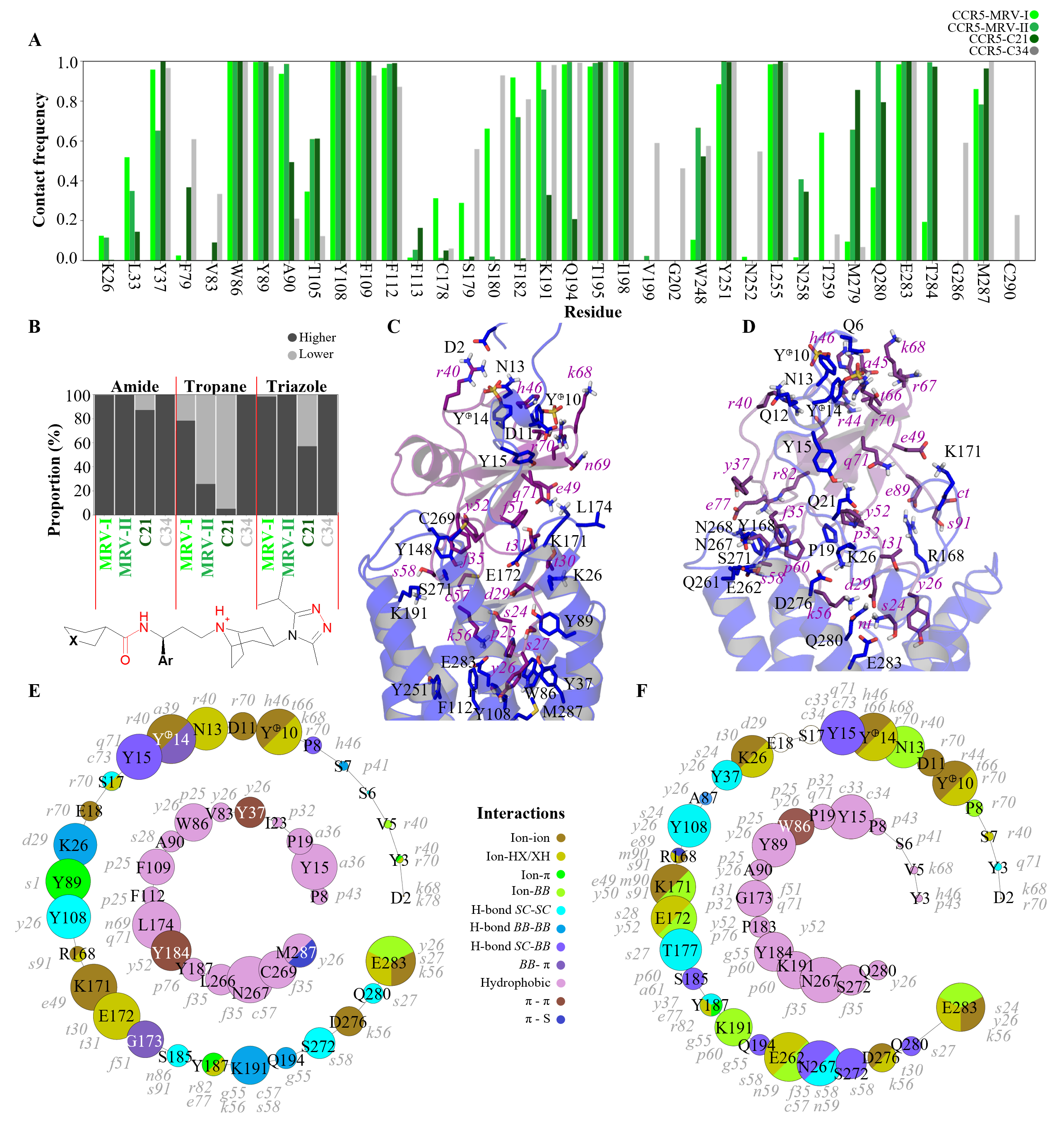

Supplement: S3 Fig — (A) Barplot of the scaled distances lesser than 4.5 Å, among the three small-molecules MRV (replicates I and II), C21 and C34 with each residue in CCR5. (B) Plot of the proportion of the distance value for the amide, tropane, and triazole moieties in the small-molecule ligands, with respect the distance in the experimental structures. (C) Interactions of CCL5 in the CCR5 representative conformer of the replicates I and II. The chemokine residues are in lowercase. (D) Plot of the interaction types in the CCR5-CCL5 complex. The inner ring counts for the hydrophobic and the outer for the polar interactions between the receptor and the chemokine. Each color represents the type of interaction, and the relative size of the circles indicates the closeness of the CCR5 residue to CCL5. (TIF) [file pone.0275269.s003.tif]

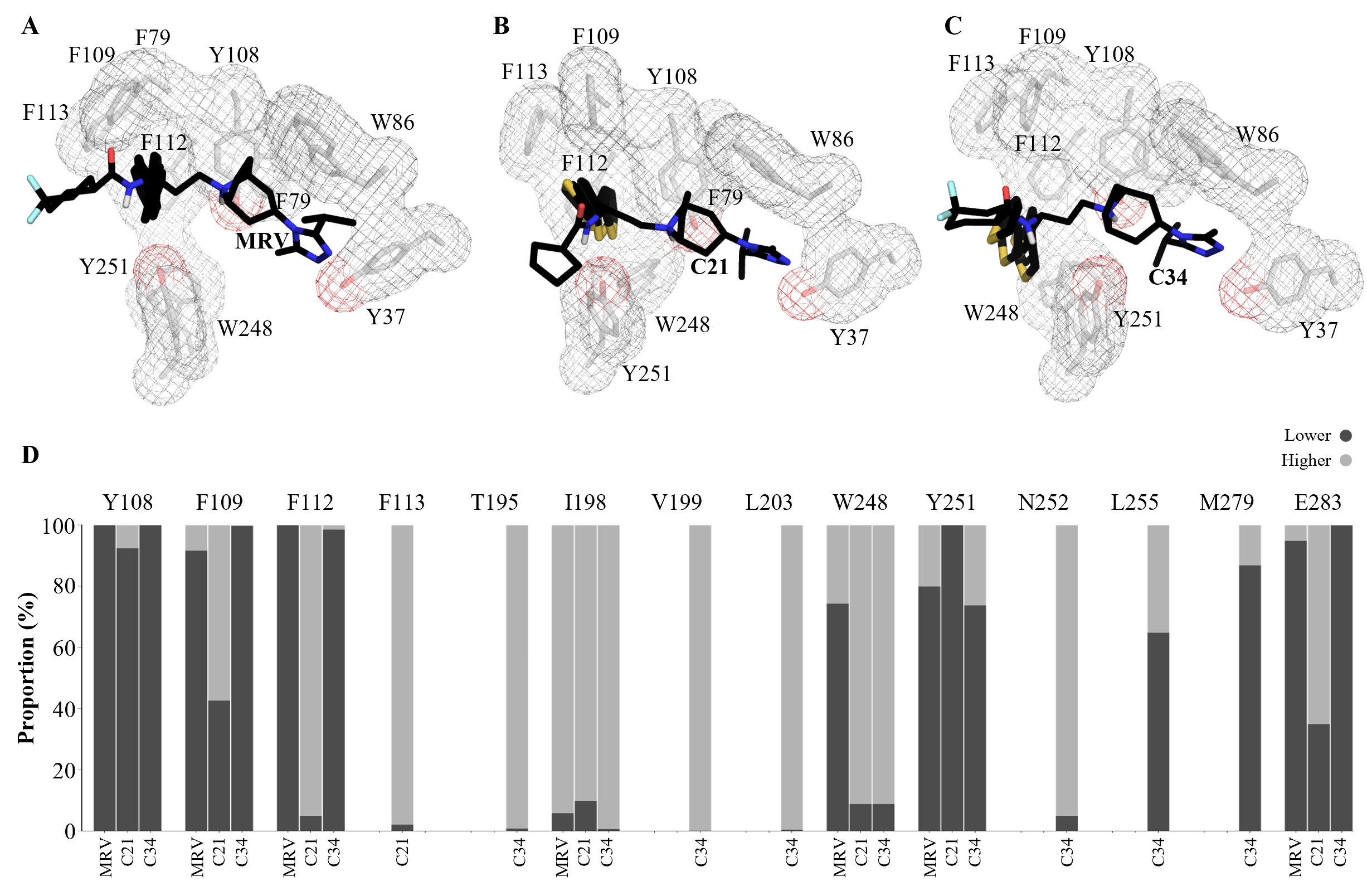

Supplement: S4 Fig — (A) MRV, (B) C21 and (C) C34. The three ligands are positioned with the arene ring towards HL1, and for MRV and C21, surrounded by it, particularly F109 and F113 at the TM3 side, W248 and Y251 at TM6 side, and F112 at a hydrophobic pocket among TM3, TM5 and TM6. For C34, the thienyl ring are displaced from the hydrophobic environment, and exposed to the interhelix side of TM5 and TM6. (D) Clustered barplots, grouped by small-molecule ligand systems, indicating the proportion of higher or lower distance from each residue in CCR5 to the arene moiety, with the distance from experimental structures as reference. MRV phenyl ring only exhibits large distancing from I198, whilst comes near to the rest of the residues. C21 shows distancing from all the residues excepting Y251, compared with the other two ligands. C34 establishes contact with several residues that MRV and C21 do not, such as T195, V199, L203, N252, L255, and M279, being the two later the closer residues than in the experimental complex. Some residues are in contact with the small-molecule ligands only in one or two systems, and in a larger distance in all of these cases. (TIF) [file pone.0275269.s004.tif]

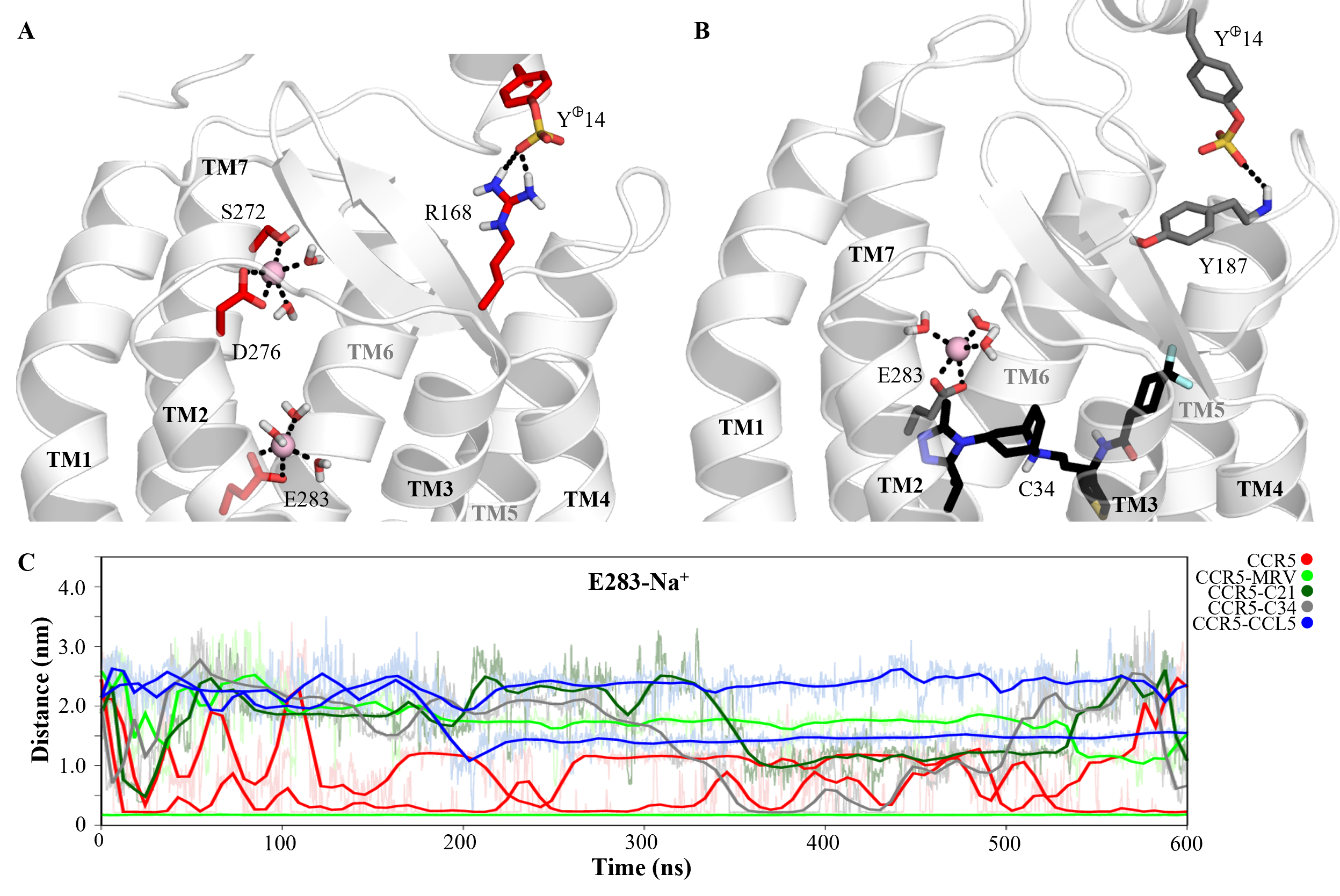

Supplement: S5 Fig — (A) apo-CCR5 (replicate I), and (B) CCR5-C34 systems. D276 and S272 coordinate a Na+ in apo-CCR5 system. Tyrosine sulfate residue (Y14) are not available to coordinate sodium cations since it interacts with either (A) R168 or (B) Y187 backbone. (C) Minimal distance of E283 carboxylate to any Na+. (TIF) [file pone.0275269.s005.tif]

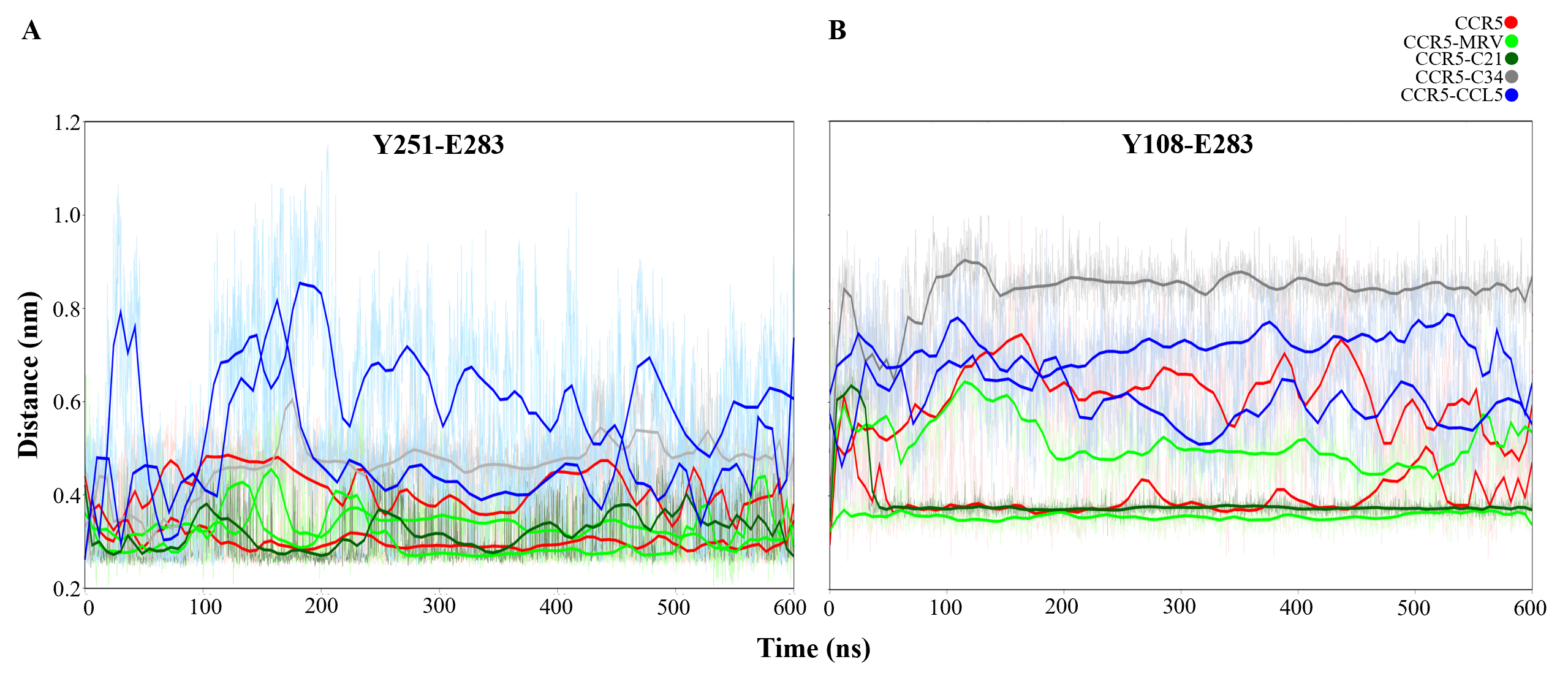

Supplement: S6 Fig — Distances from (A) Y251 and (B) Y108. In CCR5-CCL5 system, Y251 moves away from E283 to W248 vicinity in a greater extent than the other systems, and lesser in CCR5-MRV and CCR5-C21. Y108 remains closer to E283 in CCR5-C21 and apoCCR5, whilst it displaces farther in CCR5-CCL5 and CCR5-C34, suggesting that the chemokine, solely structural difference is not responsible of the separation of that pair. (TIF) [file pone.0275269.s006.tif]

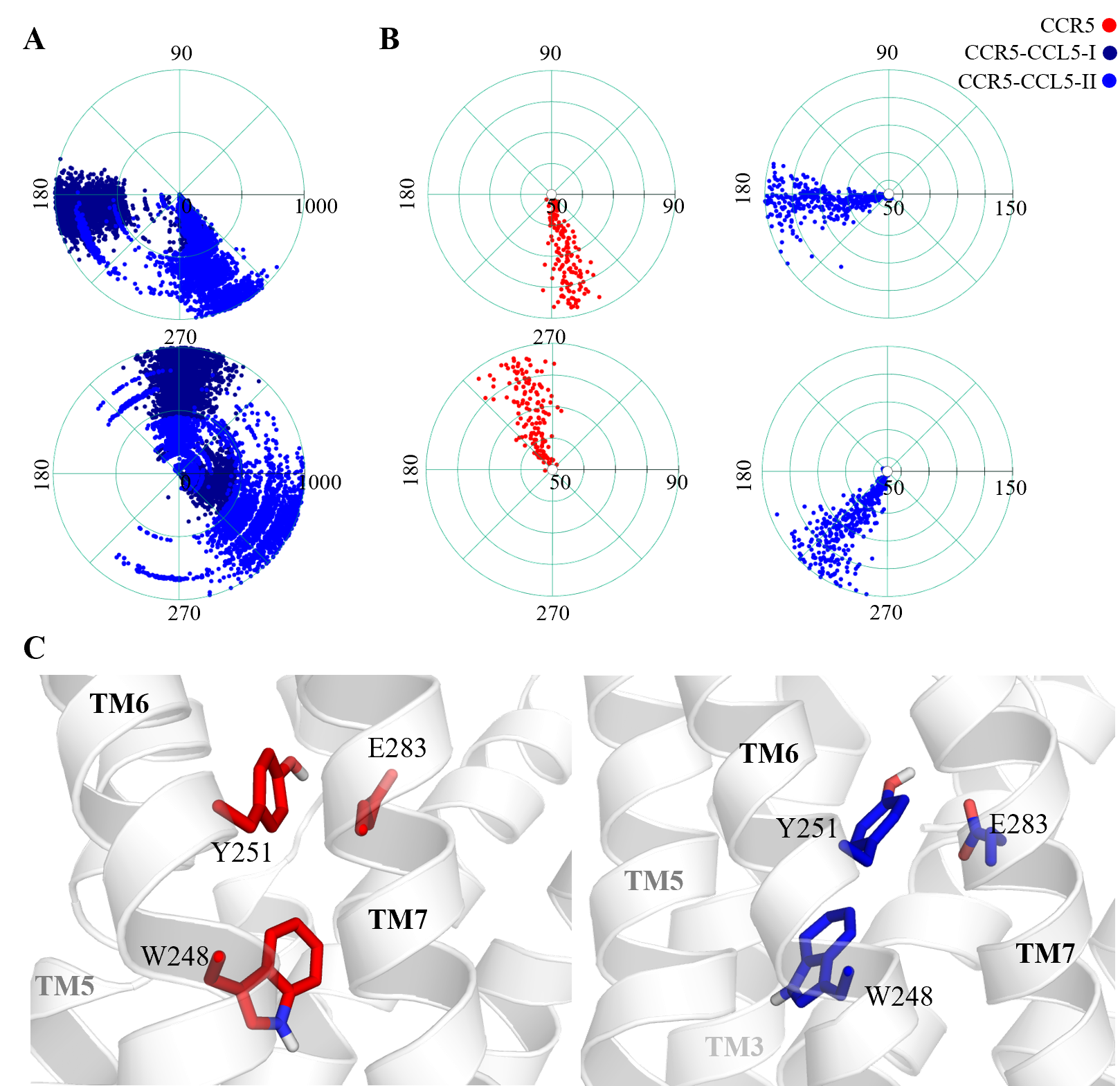

Supplement: S7 Fig — (A) From the 1 μs extended simulation of CCR5-CCL5-I, where the torsion reached in the short version of 600 ns keeps. (B) From the GaMD systems for apoCCR5 and CCR5-CCL5 systems. Both torsion angles for apo-receptor are consistent with the cMD simulation before the change at 230 ns, suggesting the lack of a change of state in the transmission switch. For chemokine-bound receptor, the χ1 angle reaches the same configuration of the cMD at 440 ns, and the χ1 angle assumes a different value, but (C) a configuration of the indole sidechain oriented to TM5 and water pore as in the cMD system of CCR5-CCL5-II. In the apo-receptor system the configuration is quite similar than the cMD simulation. (TIF) [file pone.0275269.s007.tif]

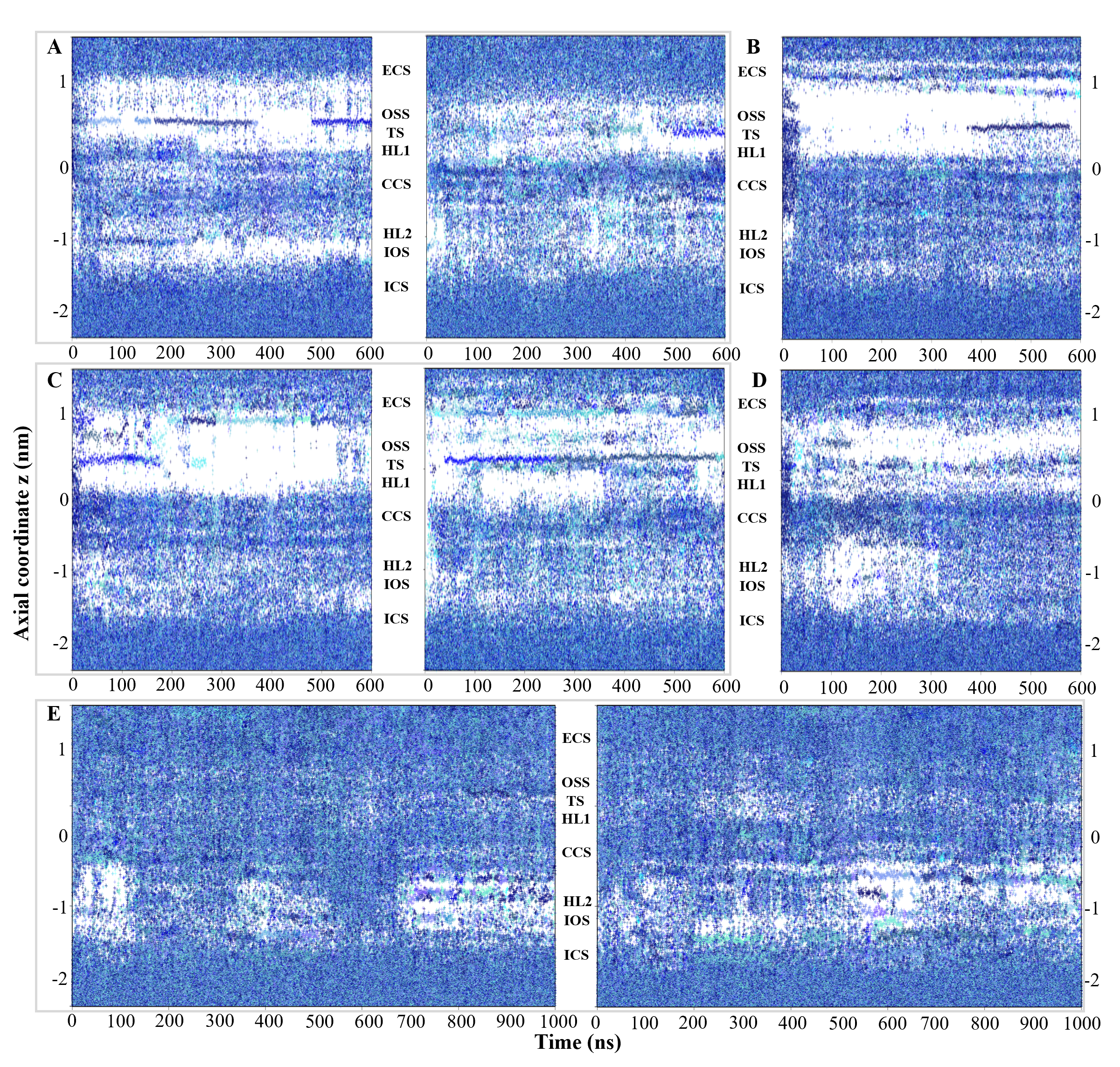

Supplement: S8 Fig — (A) apo-I and II, and small-molecule ligand systems (B) C21, (C) MRV-II and -II, (D) C34, and (E) CCL5-I and -II. HL1 and CCS regions are similar in the four systems, with a slightly more hydrated pattern in CCR5-C34. The apo-CCR5 system shows a narrow, constant dehydrated region in HL2, whilst the MRV, C21 and C34 present variable patterns in this region. C21 system was more hydrated at HL2, and C34 complex displays a broad dehydrated region between CCS and HL2 for about 300 ns of the simulation. (TIF) [file pone.0275269.s008.tif]

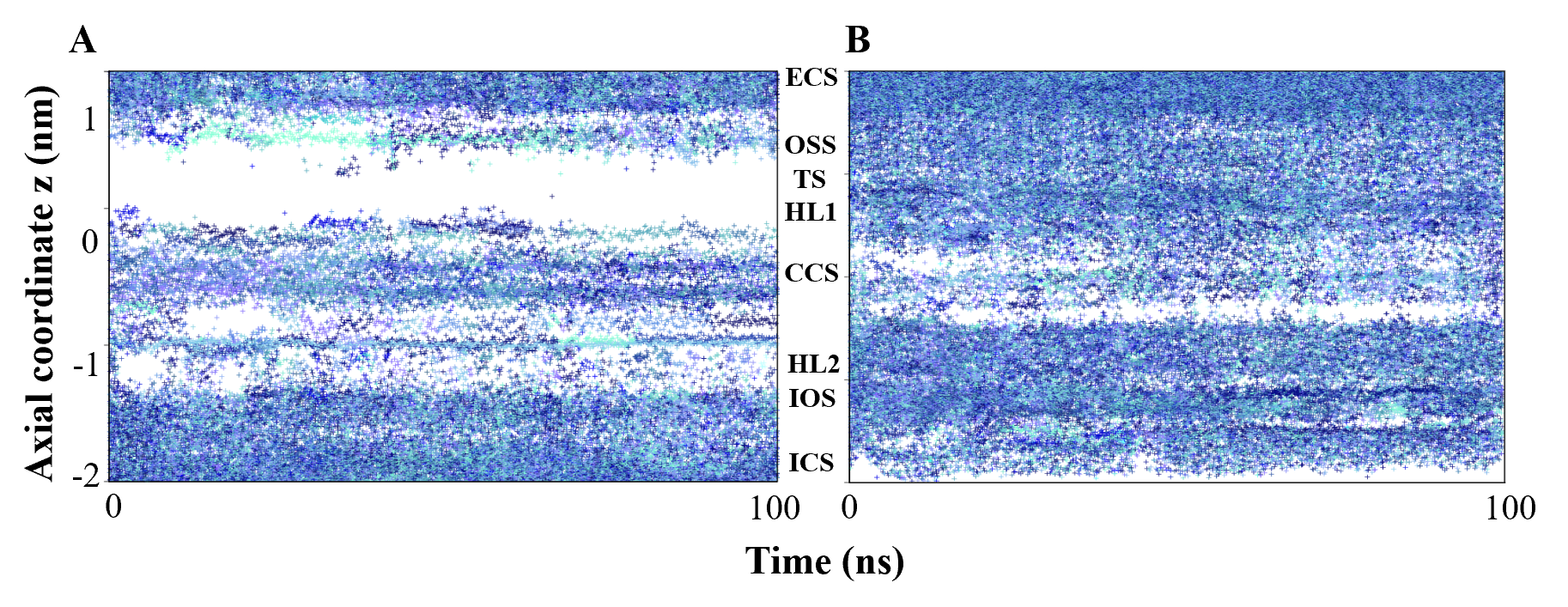

Supplement: S9 Fig — (A) apo-CCR5 and (B) CCR5-CCL5 systems. In the prior the dehydrated zone remains and, in the latter, a hydrated pattern like the cMD simulation is reached since the beginning. (TIF) [file pone.0275269.s009.tif]

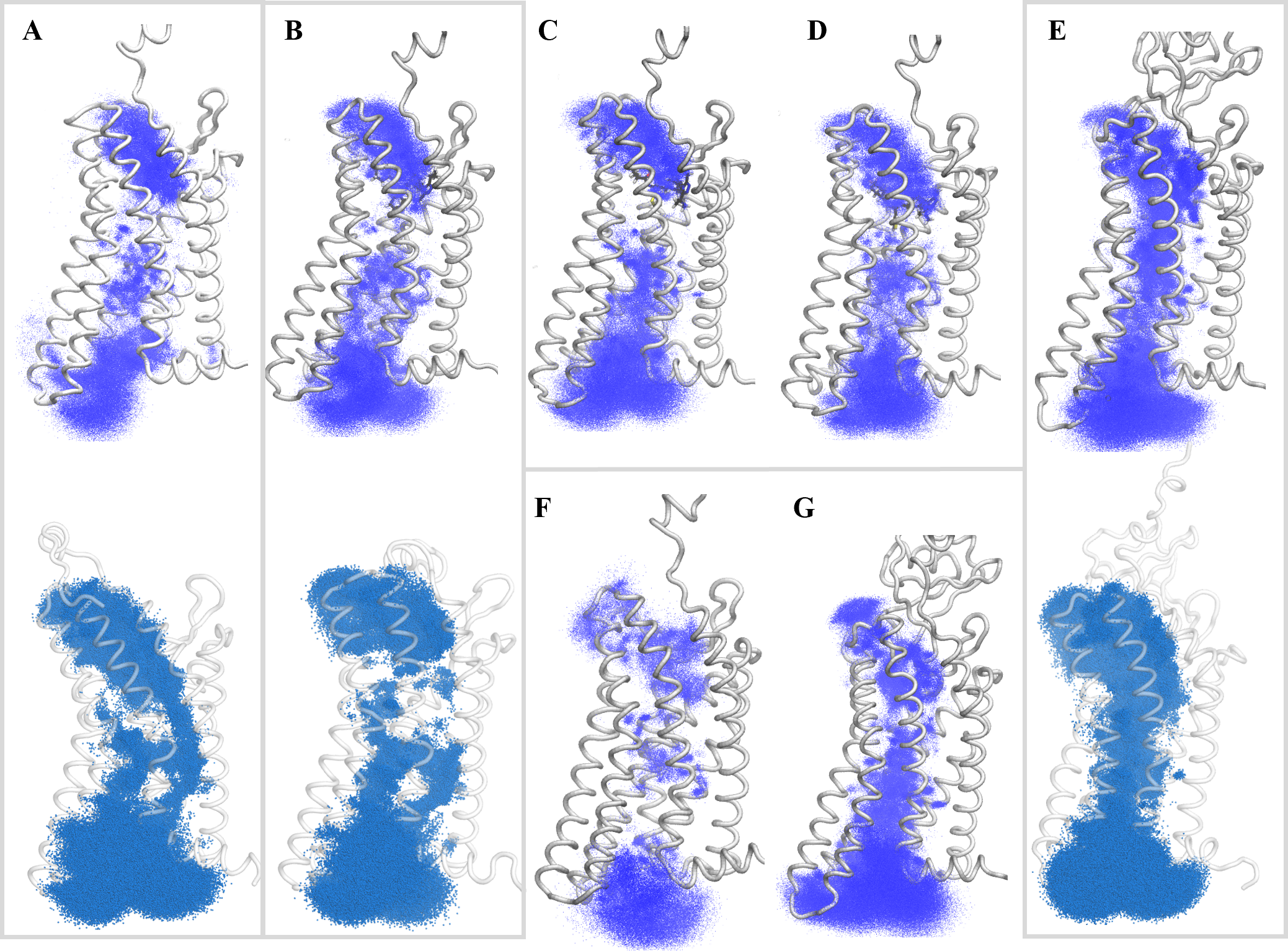

Supplement: S10 Fig — For the cMD simulations: (A) apo-CCR5-I and -II, (B) CCR5-MRV-I and -II, (C) CCR5-C21, (D) CCR5-C34, and (E) CCR5-CCL5-I and -II. For the GaMD simulations: (F) apo-CCR5 and (G) CCR5-CCL5 systems. In the non-active systems is evident the interruption of the water continuous path in the HL1 mainly, and in a lesser extent in HL2, but in the accelerated simulation. In both sampling schemes, the chemokine-bound systems possess a continuous water path overall the interhelix pore. (TIF) [file pone.0275269.s010.tif]

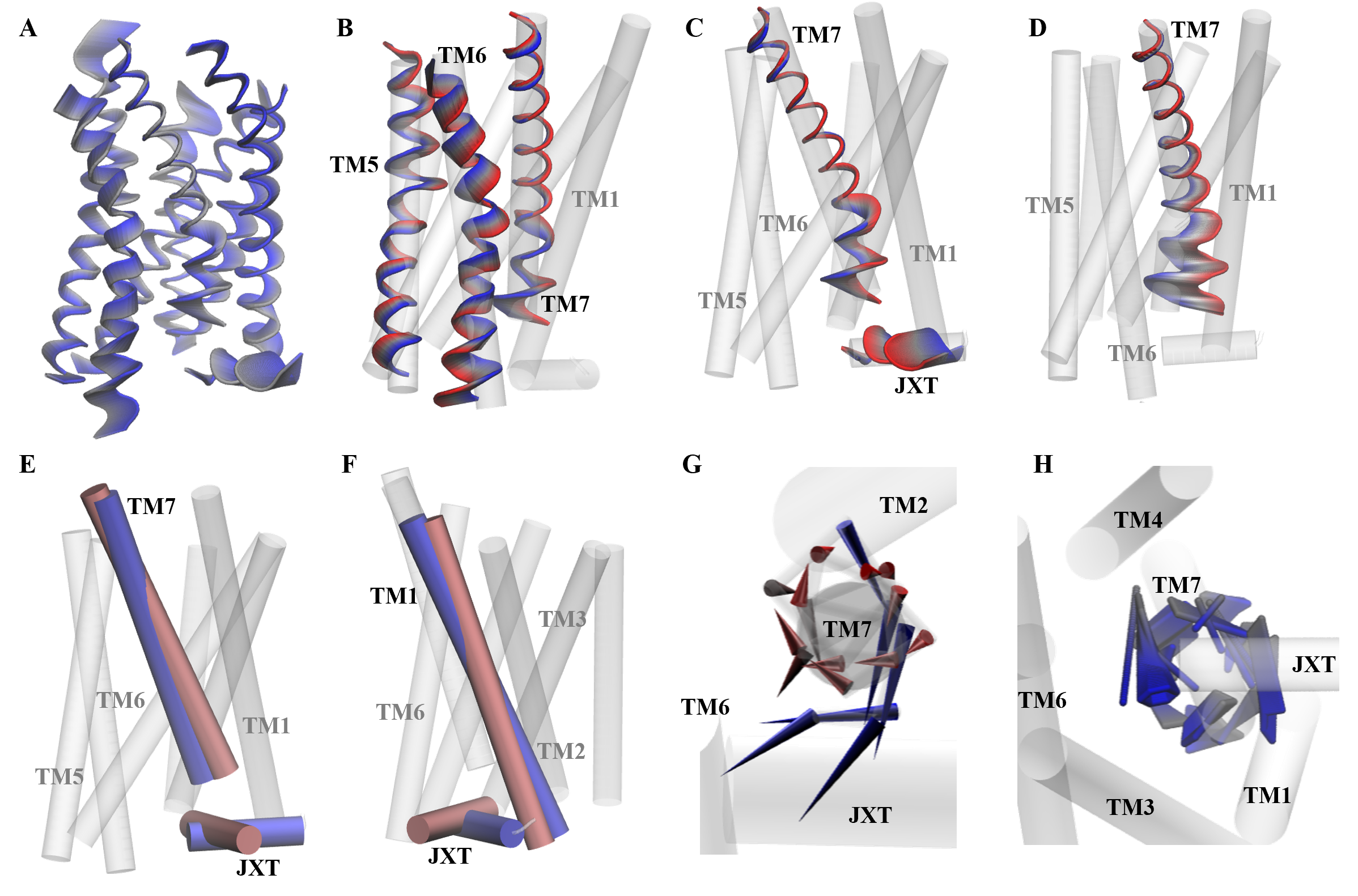

Supplement: S11 Fig — The motion begins in the reddish and gray colors and ends in blue color. (A) Principal mode 1 (PC1) of TMD, representing the % of the total essential variance of motion. (B) PC1 within TM5, TM6 and TM7 helices, that shows the distinctive motions between the EC and the IC part of each helix. The EC part of TM5 make a left-handed gyration, whilst the IC part makes a displacement towards TM6. The EC part of TM6 moves away the TMD and the EC region, and the IC segment makes a scissoring bend to TM7 and to the upper direction. The IC part of TM7 displaces to TM6 with a torsion. (C) H8 and its adjacent TM7 portion make an anti-symmetrical stretching, whereas H8 moves to TM1, displacing it. (D) The PC2 of TM7shows a larger motion of their IC part to the inter-helix pore. (E) The global helix motions in PC1 of TM7 shows the anti-symmetrical motion with H8, the prior towards the pore and the later to TM1, where (F) it is displaced by H8 to TM2. (G) The gyration of the IC part of TM7 is right-handed, as is evident with the porcupine representation of the backbone. (H) The sidechains of the same region gyrate like the backbone, excepting Y297, whose sidechain moves to the other direction. Thus, this contrary motion of Y297 let the sidechain remaining to the pore. (TIF) [file pone.0275269.s011.tif]

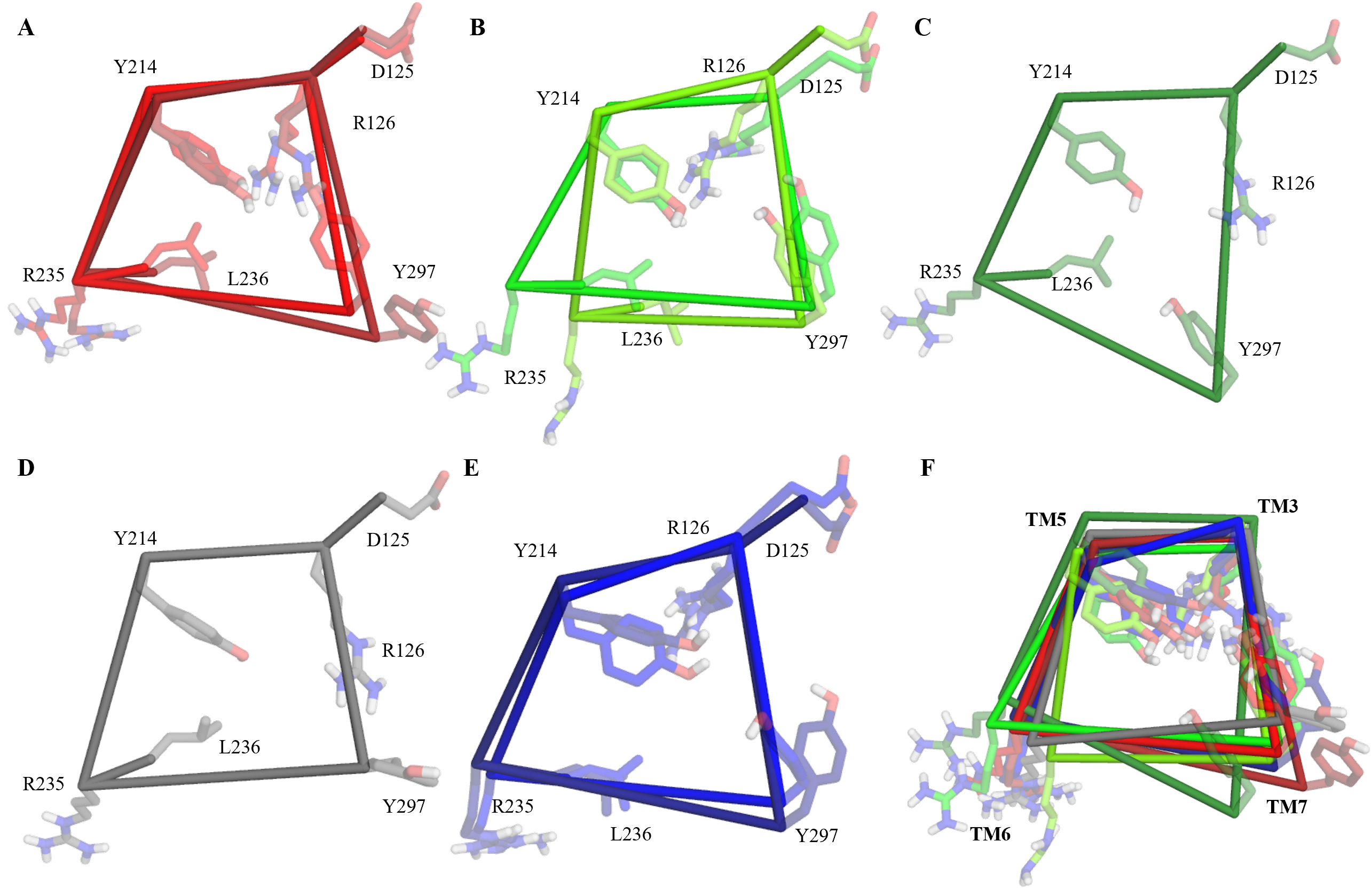

Supplement: S12 Fig — D1253.49, R1263.50, Y2145.58, R2356.35, Y2977.53, and L2366.36, for (A) apo-CCR5, (B) CCR5-MRV, (C) CCR5-C21, (D) CCR5-C34, (E) CCR5-CCL5, and (F) all systems. The quadrilateral labels in gray color represent the average distance among the Cα atoms at TM3, TM5, TM6 and TM7 respectively. The notable features are the long distances between 1: R126 and Y214 in CCR5-MRV; 2: Y214 and R235 in CCR5-MRV; 3: R235 and Y297 in apo-CCR5, CCR5-C21 and CCR5-CCL5; and 4: Y297 and R126 in CCR5-C21. The rotamers that lay the sidechain of L236 towards the pore are frequent in the apo- system, and in the complexes with C21 and C34, whilst in those with MRV and CCL5 clear the pore of the sidechain. (TIF) [file pone.0275269.s012.tif]

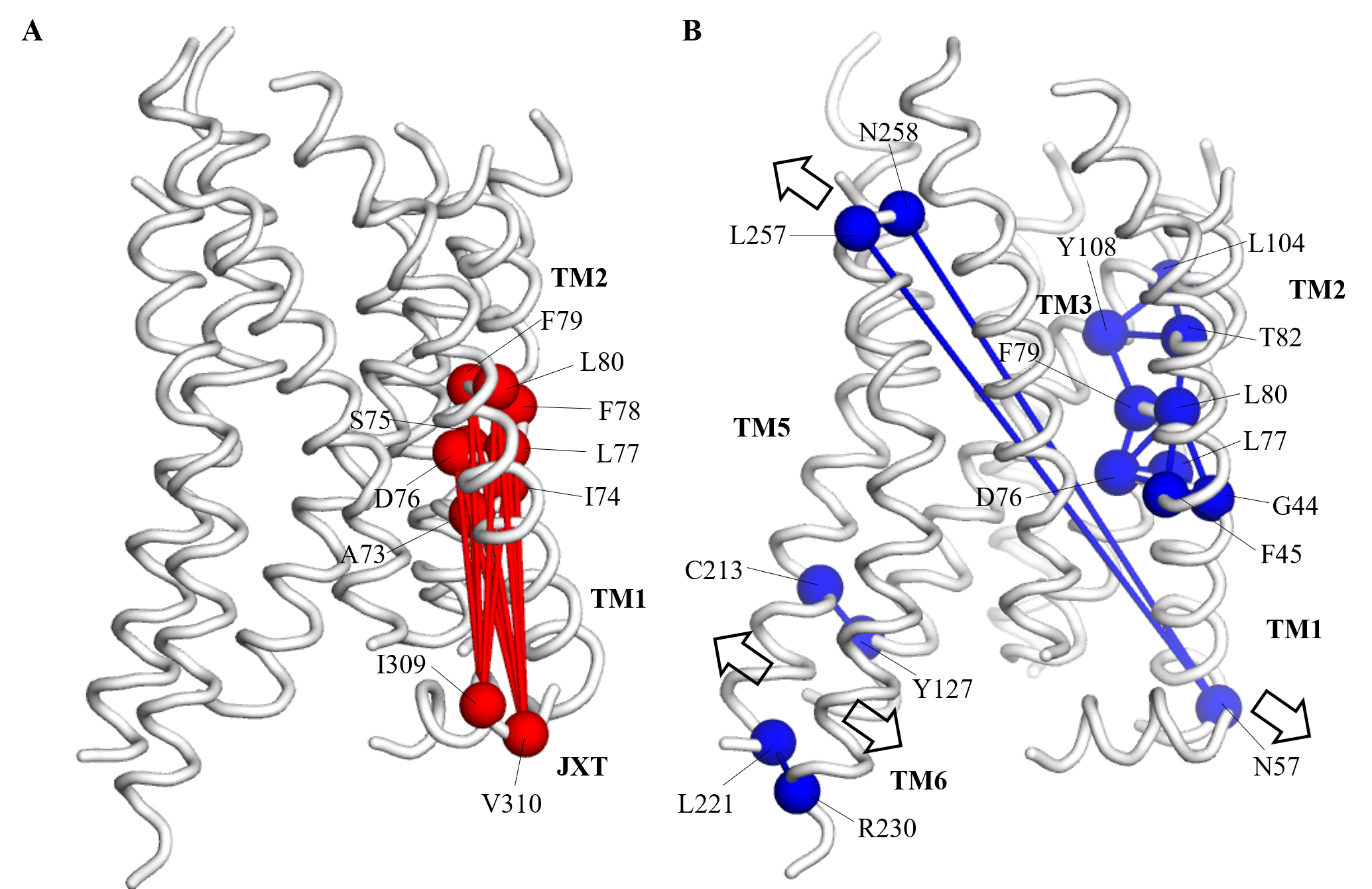

Supplement: S13 Fig — The significative residue network in TMs is for (A) positive cross-correlation within 0.6 to 0.8, and (B) negative cross-correlation within -0.6 to -0.8. The positive cross-correlation in the concerted motion is centered in TM1, TM2 and H8, from the CCS to the IC zone, related with the principal mode 1 in this region. The negative cross-correlation motions involve residues of TM1, TM2 and TM3, for CCS to the EC side. Also, two residues in in TM6-EC and one in TM1-IC, suggesting the opening of the pore and the acquisition of a trapezoidal form of the receptor. Between TM5 and TM6, at the IC side, two paired zones are correlated, involving Y127 of the DRY motif, and the distal part of both helices in the IC face, suggesting a scissoring bend between TM5 and TM6. (TIF) [file pone.0275269.s013.tif]

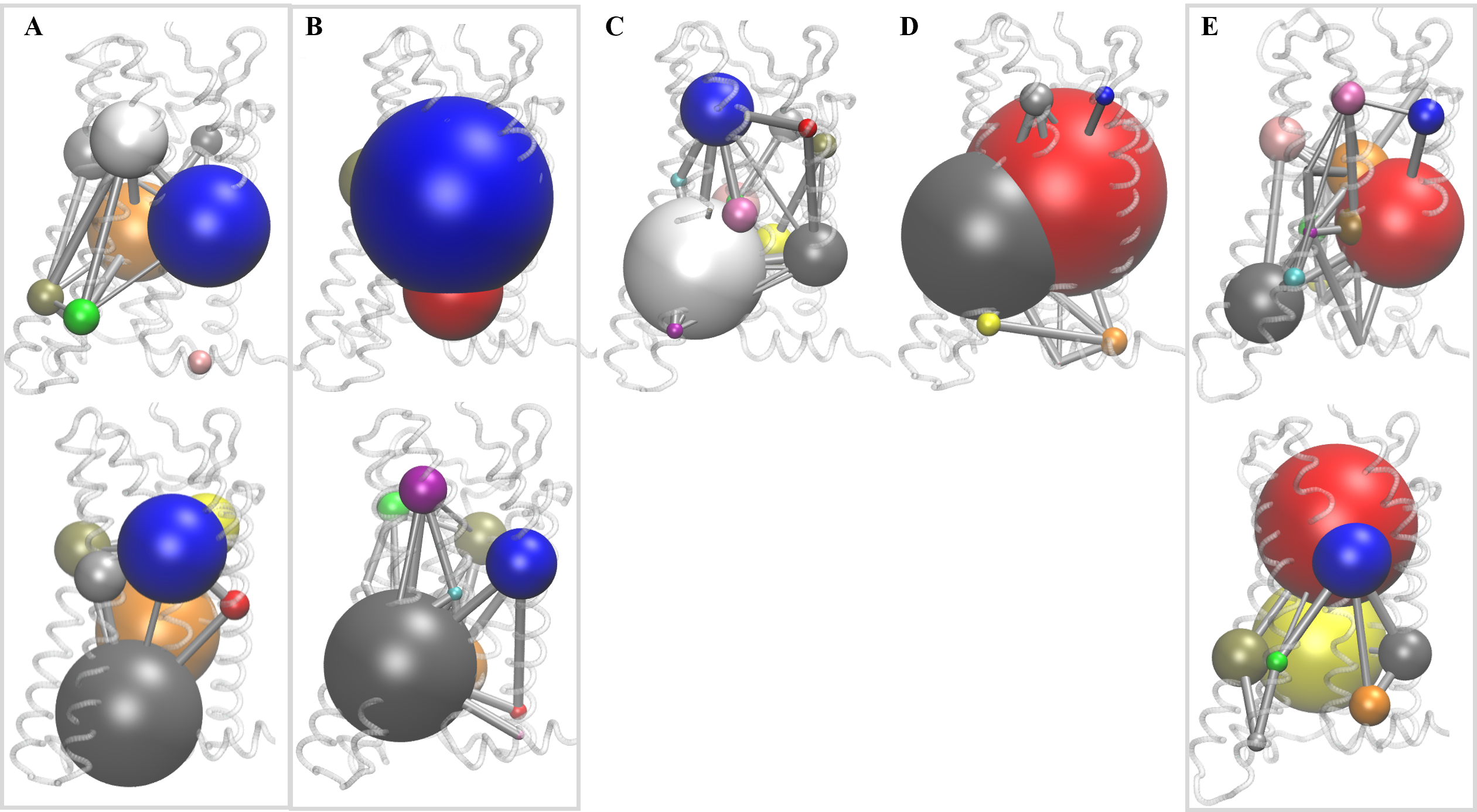

Supplement: S14 Fig — Each community represents a large, adjacent Pearson cross-correlation among the Cα atoms for (A) apo-CCR5, and (B) CCR5-MRV system; (C) CCR5-C21, (D) CCR5-C34, and (E) CCR5-CCL5 systems. The color of each sphere indicates the high correlation within (in descendent order: blue, red, dark gray, orange, yellow, light gray, green, white, pink, cyan). The relative size is related with the extension and ponderation of the positions of every correlated member in the community. It is noticeable that the CCR5-MRV and CCR5-C34 systems show extensive communities (first and second respectively) through the TMD region. In contrast, the apo-CCR5, CCR5-C21 and CCR5-CCL5 exhibit very partitioned patterns overall the communities, suggesting a perturbation that may be related with the water dynamics within the interhelix pore. (TIF) [file pone.0275269.s014.tif]

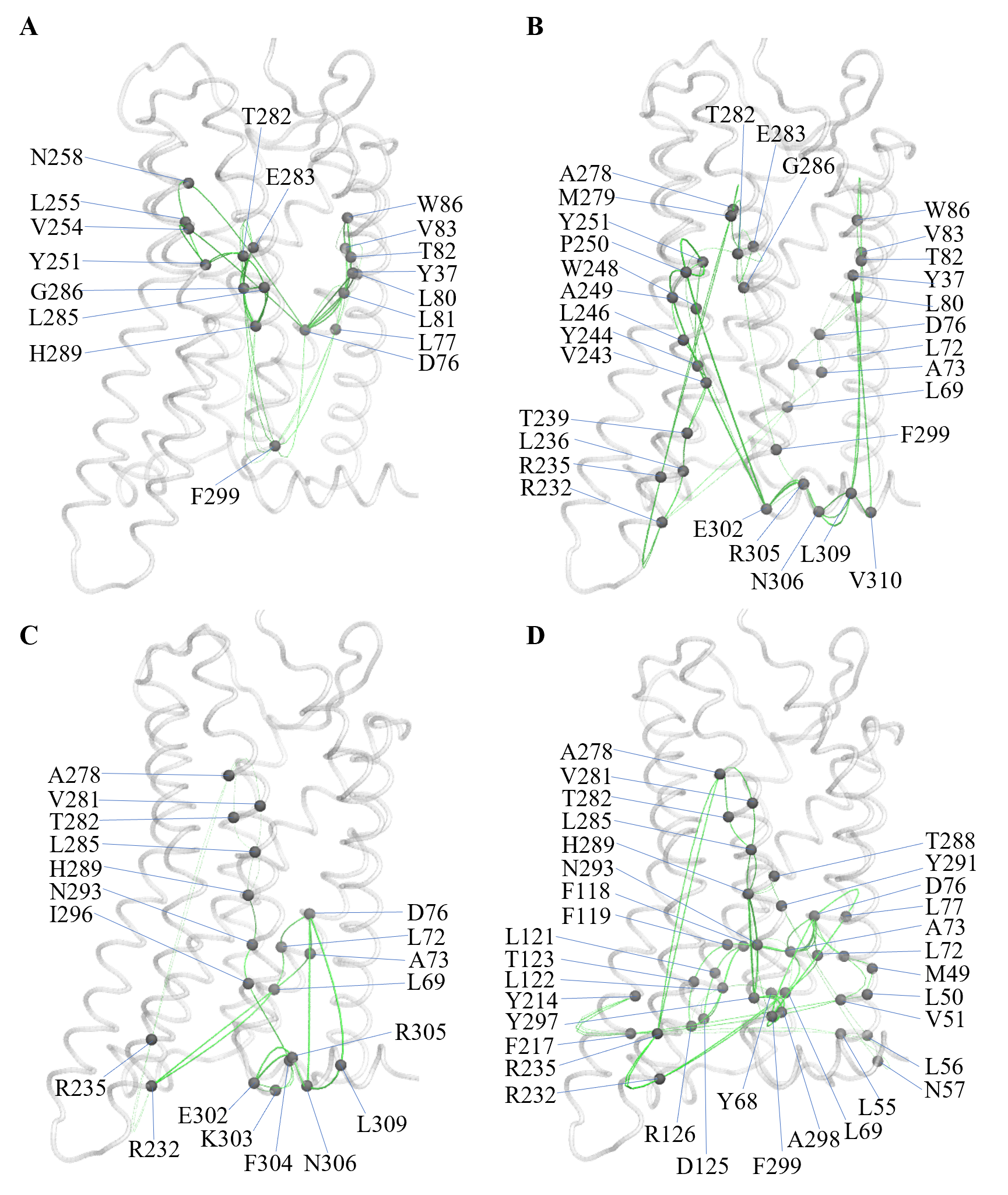

Supplement: S15 Fig — (A) Orthosteric site and transmission switch, (B) orthosteric site and ionic lock region, (C) transmission switch and central coordination site, and (D) transmission switch and R232-E302 ionic pair. (TIF) [file pone.0275269.s015.tif]

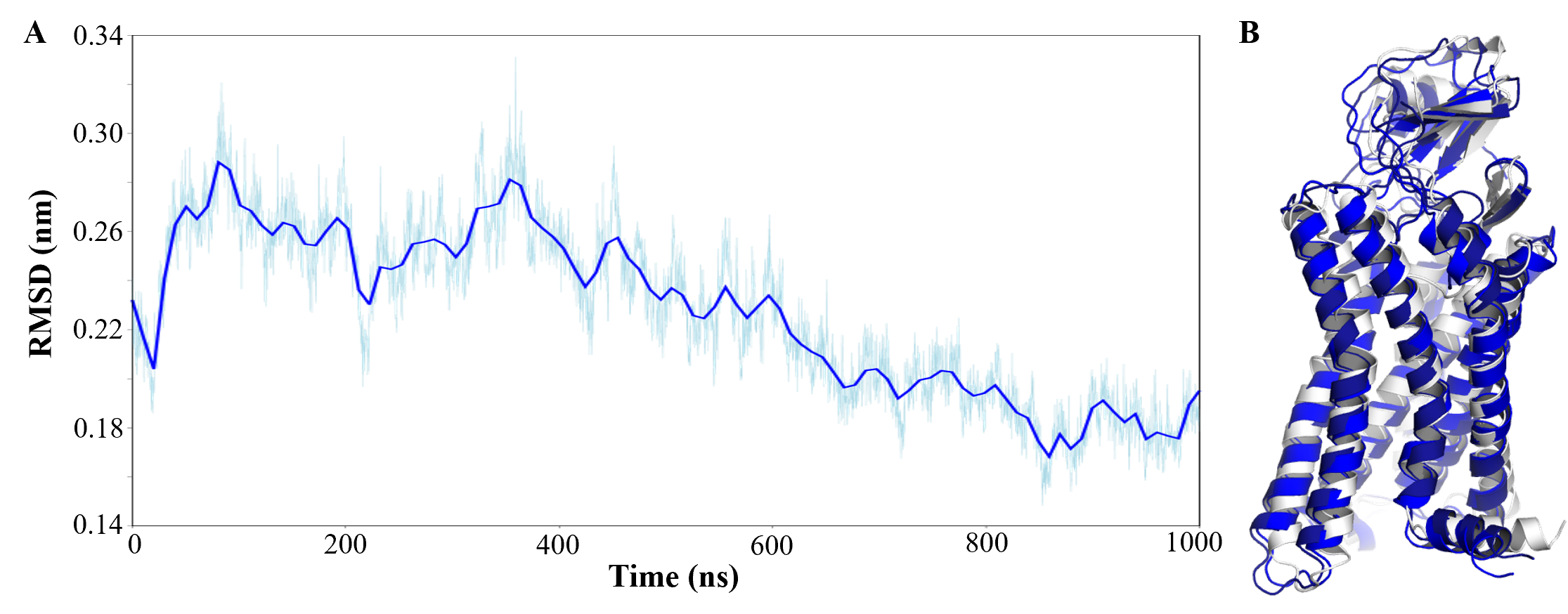

Supplement: S16 Fig — (A) The RMSD of the backbone with a defined secondary structure (i.e., helices and beta-sheets) were calculated on our trajectory with respect 7O7F. The predominant decrease in the RMSD of replicate I suggests that our system approximates to the full activated state, despite the bias introduced by the CCL5 mutations in 7O7F. (B) Superposition of both complexes, our simulated system (blue color) and the experimental system (light gray color). (TIF) [file pone.0275269.s016.tif]

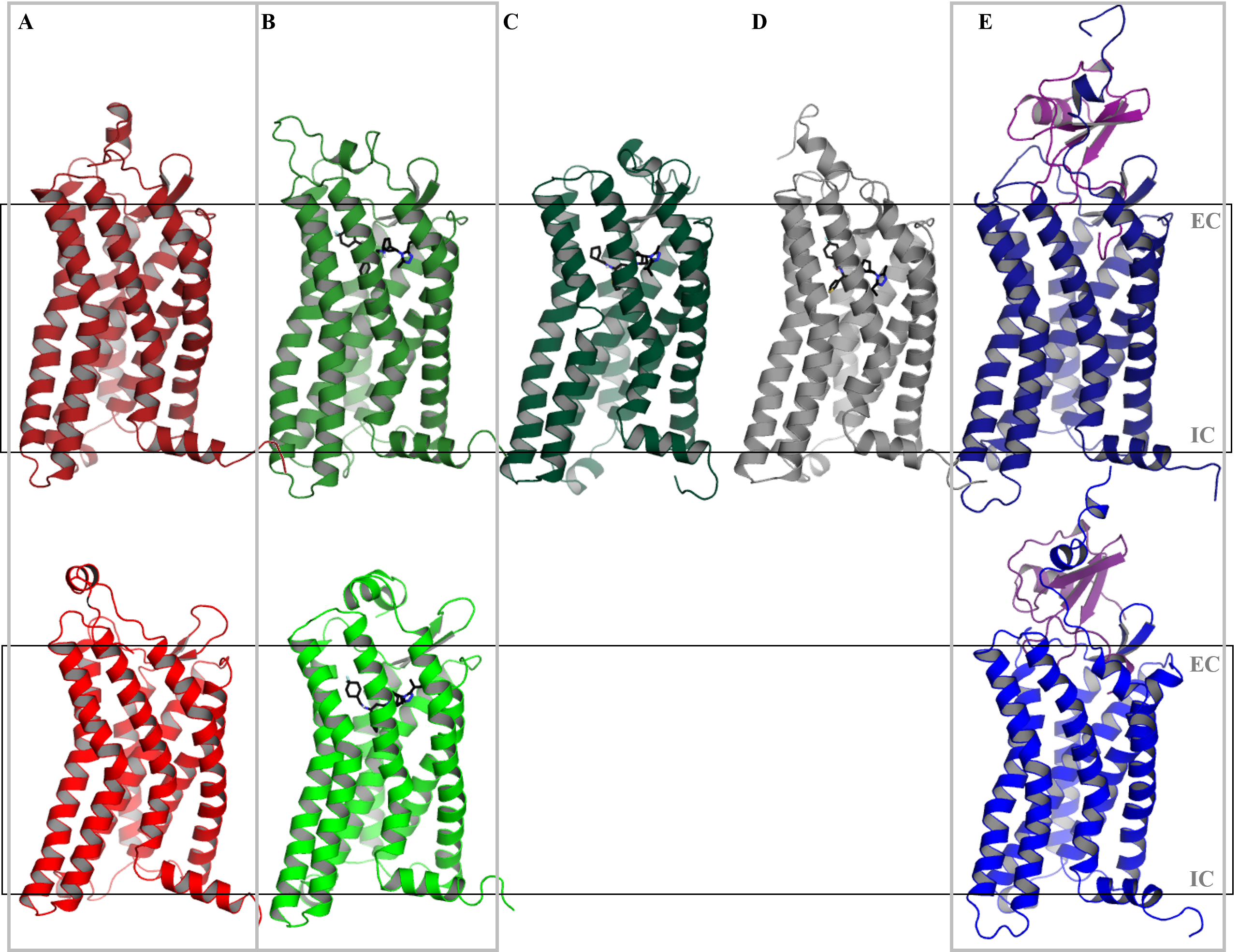

Supplement: S17 Fig — (A) apo-CCR5, (B) CCR5-MRV, (C) CCR5-C21, (D) CCR5-C34 and (E) CCR5-CCL5. The main differences are the barrel-shaped geometry of the inactive conformer and the separation of the IC extremes of TM6 and TM7. (TIF) [file pone.0275269.s017.tif]

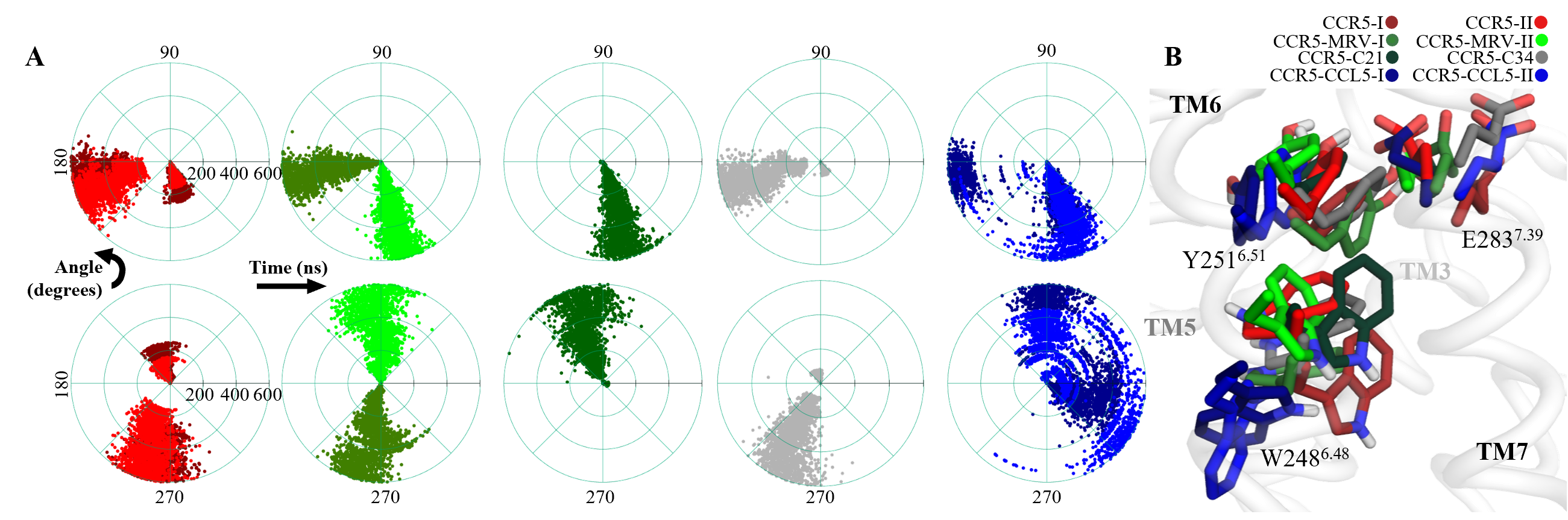

Supplement: S18 Fig — (A) apo-system, and complexes with MRV, C21, C34 and CCL5. (B) Configuration of the sidechain of W2486.48 in the backbone-aligned clustered structures. (TIF) [file pone.0275269.s018.tif]

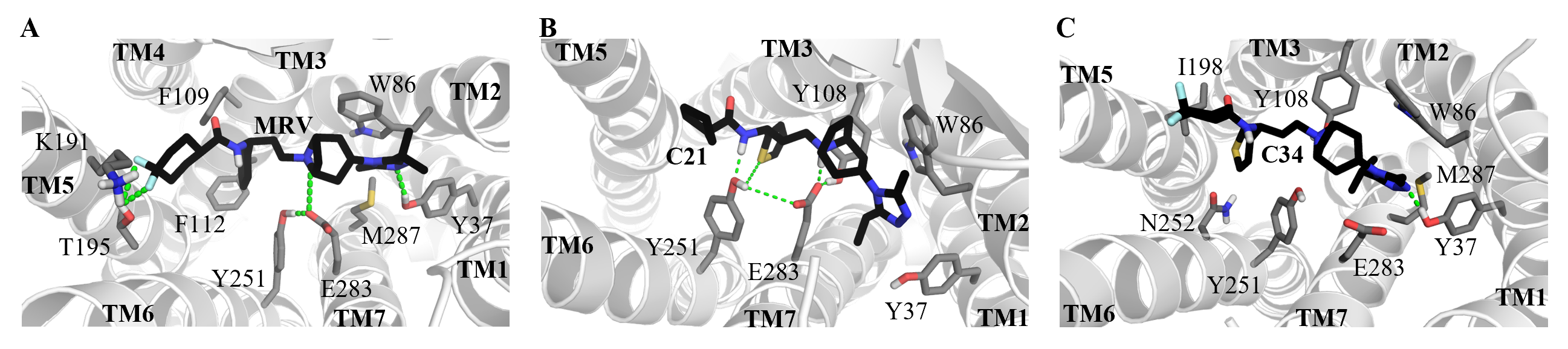

Supplement: S19 Fig — (A) MRV, (B) C21 and (C) C34 in the cavity of the orthosteric site. (TIF) [file pone.0275269.s019.tif]
